# Mapping ADHD Heterogeneity and Biotypes by Topological Deviations in Morphometric Similarity Networks

**DOI:** 10.1001/jamapsychiatry.2026.0001

**Published:** 2026-02-25

**Authors:** Nanfang Pan, Yajing Long, Kun Qin, Isaac Z. Pope, Qiuxing Chen, Ziyu Zhu, Ying Cao, Lei Li, Manpreet K. Singh, Robert K. McNamara, Melissa P. DelBello, Ying Chen, Alex Fornito, Qiyong Gong

**Affiliations:** 1 Department of Radiology, Huaxi MR Research Center, Institute of Radiology and Medical Imaging, Psychoradiology Key Laboratory of Sichuan Province, Research Unit of Psychoradiology, Chinese Academy of Medical Sciences, West China Hospital of Sichuan University, Chengdu, China; 2 The Turner Institute for Brain and Mental Health, School of Psychological Sciences and Monash Biomedical Imaging, Monash University, Clayton, Victoria, Australia; 3 Department of Radiology, Taihe Hospital, Hubei University of Medicine, Shiyan, China; 4 Department of Psychiatry, University of Cincinnati, Cincinnati, Ohio; 5 Department of Psychiatry and Behavioral Sciences, University of California, Davis, Sacramento; 6 Mental Health Center, West China Xiamen Hospital of Sichuan University, Xiamen, China; 7 Xiamen Key Lab of Psychoradiology and Neuromodulation, Department of Radiology, West China Xiamen Hospital of Sichuan University, Xiamen, China

## Abstract

**Importance:**

Attention-deficit/hyperactivity disorder (ADHD) is characterized by considerable clinical heterogeneity, and existing classification frameworks constrain the development of neurobiologically informed subtyping approaches.

**Objective:**

To investigate whether normative modeling of topological properties derived from brain morphometry similarity networks can provide robust stratification markers for children with ADHD.

**Design, Settings, and Participants:**

This case-control study leveraged multisite cross-sectional neurodevelopmental datasets with a longitudinal follow-up cognitive assessment for a subset. Morphometric similarity networks were constructed and normative models were developed for 3 topological metrics: degree centrality, nodal efficiency, and participation coefficient. Through semisupervised clustering, putative biotypes were delineated and their clinical profiles were examined. Brain profiles of these biotypes were further contextualized in terms of their neurochemical and functional correlates using large-scale databases, and model generalizability was assessed with external validation performed in an independent transdiagnostic cohort. Study data were analyzed from November 2023 to January 2025.

**Exposures:**

Normative modeling of topological properties derived from brain morphometry.

**Main Outcomes and Measures:**

Topological deviations in morphometric similarity networks derived from brain structural image.

**Results:**

The discovery cohort comprised 446 children with ADHD (mean [SD] age, 11.5 [2.6] years; 339 male [76.0%]) and 708 controls (mean [SD] age, 11.0 [2.3] years; 429 male [60.6%]), whereas the validation cohort included 554 children with ADHD (mean [SD] age, 10.1 [2.8]; 372 male [67.1%]) and 123 controls (mean [SD] age, 10.1 [3.0]; 70 male [56.9%]). ADHD exhibited atypical hub organization across all 3 topological metrics, with significant case-control differences primarily localized to a covarying multimetric component in the orbitofrontal cortex. Three biotypes emerged: severe-combined with emotional dysregulation (widespread medial prefrontal cortex-pallidum alterations, n = 142), predominantly hyperactive/impulsive (anterior cingulate cortex-pallidum circuit alterations, n = 177), and predominantly inattentive (superior frontal gyrus alterations, n = 127), each characterized by distinct clinical profiles and longitudinal trajectories. These neural profiles of each biotype showed distinct neurochemical and functional correlates. Critically, the core findings were replicated in the validation cohort, demonstrating robust generalizability.

**Conclusions and Relevance:**

Results of this case-control study reveal that the integration of normative modeling with semisupervised clustering provided both dimensional and categorical insights into ADHD heterogeneity, identifying 3 distinct ADHD biotypes with unique clinical-neural profiles that advance the understanding of ADHD’s neurobiological complexity and lay the groundwork for personalized management.

## Key Points

**Question:**

Can normative modeling of topological properties derived from brain morphometric similarity networks yield robust stratification biomarkers for pediatric populations with attention-deficit/hyperactivity disorder (ADHD)?

**Findings:**

This multisite case-control study included 1154 participants, characterizing ADHD heterogeneity through hub-centric topological deviations derived from morphometric similarity networks. Three distinct biotypes emerged, each exhibiting unique clinical-neural profiles with characteristic neurochemical and functional correlates, validated in an independent transdiagnostic cohort of 554 ADHD cases.

**Meaning:**

The integration of normative modeling with heterogeneity through discriminative analysis (HYDRA) clustering yielded both dimensional and categorical insights into ADHD heterogeneity, thereby enhancing our understanding of the ADHD’s neurobiological complexity.

## Teaser

This case-control study investigates if normative modeling of topological properties derived from brain morphometric similarity networks yields robust stratification biomarkers for pediatric populations with attention-deficit/hyperactivity disorder (ADHD).

## Introduction

Attention-deficit/hyperactivity disorder (ADHD) is a common neurodevelopmental disorder characterized by considerable clinical heterogeneity that extends beyond the *DSM-5* diagnostic framework.^1^ Although its symptoms are partitioned into inattentive and hyperactive/impulsive domains, this binary behavioral classification inadequately captures the complexity of ADHD presentations.^2^ Clinical observations reveal diverse patterns across *DSM*-defined domains,^3^ but these consensus-based distinctions within *DSM* may oversimplify the diverse neurobiological mechanisms underlying ADHD, precluding more informative and neurobiologically homogeneous subtyping. Supervised approaches that define subtypes using checklist thresholds typically generate only severity-based cognitive subgroups.^4,5^ In contrast, data-driven clustering may offer a superior solution by leveraging phenotypic data that best informs the delineation of disorder subtypes.

There have been multiple attempts to identify ADHD subtypes by analyzing varying symptom combinations,^6^ but many fail to distinguish normative from atypical variations in phenotypic measures.^7^ Normative modeling incorporating neuroimaging metrics offers a powerful framework for overcoming this limitation and understanding atypical features,^8^ compared with case-control comparisons that rely on group contrasts and ignore individual heterogeneity. The approach is analogous to pediatric growth charts, allowing one to quantify centiles of normative variation in a given phenotype that can be used to evaluate the extent to which a given individual deviates from population expectations.^9^ Nevertheless, identifying reproducible patterns within this dimensional framework through data-driven clustering may reveal subgroups that can be used for clinical decision-making.^10^

Previous normative modeling studies in ADHD have primarily examined regional morphology,^2^ including gray matter volume,^11^ cortical thickness,^12^ and white matter volume.^13^ However, regional variations are often coupled across disparate brain systems, and the network-level coupling of deviations from normative regional morphometry remains poorly understood. Morphometric similarity networks (MSNs) can be used to characterize the covariance patterns of brain regional features, offering an individualized modeling approach with high robustness and reproducibility for identifying accessible and cost-effective magnetic resonance imaging (MRI)–derived biomarkers.^14^ Variations within MSNs can arise from a combination of interregional structural connections through axonal pathways, similarities in cytoarchitecture, and shared patterns of gene expression.^15^ These networks often exhibit hubs—specific regions that are considered particularly influential in brain networks by virtue of their coupling patterns.^14^ Multiple measures exist for characterizing the hubness of a node within MSNs, indicating that an approach that integrates various information may be particularly fruitful.^16^ Such approaches provide a promising framework for understanding brain network alterations in neurodevelopment with robust sensitivity^17^ and may also serve as neurobiological markers for ADHD.^18^

We therefore hypothesized that (1) normative modeling of MSN hubness would reveal heterogeneity in children with ADHD, (2) data-driven clustering of these multimetric hubness deviations would identify distinct neurobiological biotypes with unique clinical-biological profiles, and (3) these biotypes could be contextualized with respect to distinct neurotransmitter density maps and functional correlates.

## Methods

Our discovery project analyzed multisite data from West China Hospital of Sichuan University (WCH), University of Cincinnati (UC), Kennedy Krieger Institute, New York University Langone Medical Center, Oregon Health & Science University, and Peking University Institute of Mental Health. The WCH and UC datasets received ethics approval from their respective research ethics committees. Written informed consent was obtained from all participants and their parents. This study followed the Strengthening the Reporting of Observational Studies in Epidemiology (STROBE) reporting guidelines.

### Data Collection and Morphometric Similarity Network Construction

Inclusion and exclusion criteria across sites are available in eTable 1 in Supplement 1. We also included control samples from the Autism Brain Imaging Data Exchange (ABIDE) initiative that matched the protocols of the corresponding ADHD-200 sites. To reconcile sample and cross-site heterogeneity, we excluded left-handed children and those younger than 6 years or older than 18 years. Data from a transdiagnostic database, the Healthy Brain Network initiative, were obtained as validation samples.^19^ ADHD symptom subscales were rescaled to a range of 0 to 1.0 to facilitate cross-site individual predictions.^20,21^ The majority of samples did not disclose race and ethnicity information; therefore, these data were not included in our study.

**Figure 1.**
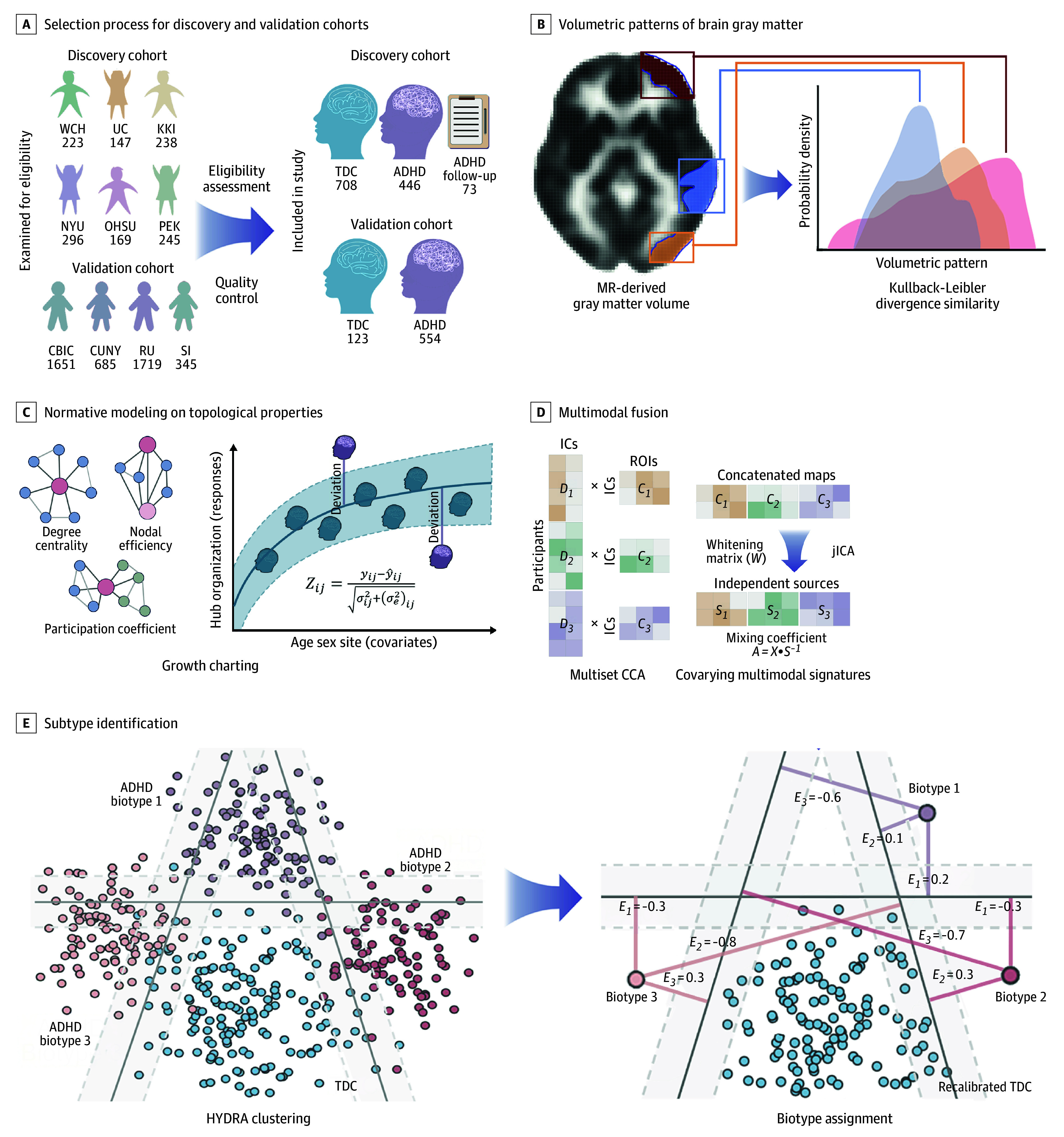
Schematic Overview of Analytical Procedures A, Structured selection process for discovery and validation cohorts. B, Volumetric patterns of brain gray matter were exacted, and morphological similarity between paired regions was quantified. C, The hub organization of morphometric similarity networks was characterized and set as responses of normative models (warped bayesian linear regression) to estimate normative centiles. D, Multimodal canonical correlation analysis (mCCA)–generated mixing profile *D_k_* and derived associated map *C_k_*, (*k* is the number of modalities) to capture intermodal associations. joint independent component analysis (jICA) was adopted on concatenated *C_k_* maps to derive maximally independent sources *S_k_* and whitening matrix W, yielding Individual-wise mixing coefficients (*A_k_*). E, Identify putative attention-deficit/hyperactivity disorder (ADHD) biotypes in heterogeneity through discriminative analysis (HYDRA) framework (simulated model), linear support vector machines were leveraged to establish multiple hyperplanes (bands between groups) that encompass the control distribution while differentiating ADHD cases. Pretrained HYDRA model was then applied to validation cohort to obtain expression scores (*E_i_*, where *i* is the optimal number of biotypes) to determine cluster assignment. KKI indicates Kennedy Krieger Institute; MR, magnetic resonance; NYU, New York University Langone Medical Center; OHSU, Oregon Health & Science University; PEK, Peking University Institute of Mental Health; ROI, region of interest; TDC, typically developing control; UC, University of Cincinnati; WCH, West China Hospital of Sichuan University.

We generated individual gray matter volume maps derived from anatomic T1-weighted images by using the Computational Anatomy Toolbox (CAT) CAT12 pipeline (Christian Gaser and Robert Dahnke) and the Statistical Parametric Mapping (SPM) SPM12 toolbox (University College London) (eMethods in Supplement 1).^22^ To quantify the morphometric similarity patterns among brain gray matter regions (Figure 1B), we used the Kullback-Leibler divergence similarity metric to assess the concordance of gray matter volume distributions between paired anatomical structures.^23^ We calculated 3 topological metrics—degree centrality, nodal efficiency, and participation coefficient to comprehensively investigate the hub organization of this network (Figure 1C); definitions and calculation processing are available in the eMethods in Supplement 1.

### Statistical Analysis

#### Normative Modeling and Multimodal Fusion Framework

Normative models for each nodal topological phenotype were implemented using the warped bayesian linear regression framework from PCNtoolkit,^8^ which estimates normative ranges for topological metrics with respect to demographic covariates (age and sex) in the training dataset (Figure 1D), using B-spline basis expansion for age and likelihood warping to accommodate non-Gaussian nonlinear effects (settings in the eMethods in Supplement 1).^24,25,26^ Individual ADHD deviations were quantified as *z *scores relative to normative centiles derived from typically developing control (TDC) training data. To assess potential site-related artifacts, we performed 2-fold linear support vector machine classification to determine whether scanning site could be predicted.^27^

Given subtle brain alterations characterized in ADHD,^27^ we identified extreme deviations in topological patterns using a threshold of |*z*| ≥2.0^28,29^ and calculated the proportion of children exhibiting atypical patterns at each node. ADHD deviation maps were then subtracted from those of TDCs to obtain maps of case-control differences in extreme deviations. To assess the statistical significance of these differences, we performed both group-based and spatial permutation tests to generate null distributions of case-control differences (details in the eMethods in Supplement 1). We identified nodes with statistically significant differences using thresholds of uncorrected *P *<.05 and false-discovery rate (FDR) *P *<.05.

To identify joint independent components that robustly encompassed covarying multimetric patterns and summarize nodal findings, topological phenotypes were subjected to a data-driven model—multiset canonical correlation analysis plus joint independent component analysis (multimodal canonical correlation analysis [mCCA] + joint independent component analysis [jICA]) (Figure 1E) using the Fusion ICA Toolbox^30,31^ (details in the eMethods in Supplement 1).

#### Biotype Identification

Heterogeneity through discriminative analysis (HYDRA), a semisupervised algorithm, was implemented to analyze individual deviations in 3 hubness measures to identify putative ADHD biotypes.^32^ It leverages support vector machines to create multiple hyperplanes that separate controls from cases to form a convex polytope, with each facet representing a distinct biotype (Figure 1F). To capture the heterogeneity of brain topological properties, we trained HYDRA models utilizing a 10-fold nested cross-validation protocol to determine the optimal *k*-dimensional space. The clustering stability was quantified through the adjusted Rand indices. The robustness of the identified ADHD biotypes was validated via 3 reproducibility analyses: permutation testing, sex-specific validation, and split-half cross-validation (analytical details in the eMethods in Supplement 1).

To investigate the corresponding clinical profiles of the ADHD biotypes, Kruskal-Wallis analysis was used to examine their differences in symptom severity.^33^ To quantify differences in atypical topological patterns among biotypes, we computed the frequency of extreme deviations within each node and used χ^2^ analyses to assess statistical differences across biotypes (uncorrected *P *<.05 and FDR *P* <.05). Biotype-specific extreme deviation maps were depicted using the same procedures described above.

To examine longitudinal changes in ADHD symptoms across biotypes, we analyzed follow-up data from the Child Behavior Checklist^34^ collected annually over 4 years at the WCH site (exclusively beginning with medication-naive participants) using linear mixed-effect modeling to examine the interaction effects of time and biotype.^35^ Additionally, we monitored the development of mood disorders (eg, anxiety and depression) among ADHD participants during follow-up.

#### Biological Correlates of Biotype Profiles

To elucidate molecular signatures underlying our topology-derived ADHD biotypes, we characterized their correspondence to the spatial distributions of neurotransmitter receptors (Figure 1G).^36^ The neurotransmitter receptor maps encompass density distributions of 19 distinct receptors derived from positron emission tomography imaging studies (eTable 2 in Supplement 1 and analytical procedures in the eMethods in Supplement 1).^37^ To comprehensively characterize brain network organization of ADHD, we aggregated case-control difference maps in extreme deviations across 3 topological metrics (termed as fused topological deviations)^38^ and then used Spearman correlation to quantify the spatial correspondence with neurotransmitter receptor distributions with spin tests (ie, spatial permutation tests) assessing statistical significance (5000 surrogates).^39^

We used the Neurosynth-based meta-analytic task activation maps to contextualize our findings beyond the available clinical measures and to explore associations with a broader spectrum of psychological processes.^40^ Neurosynth Compose,^41^ an automated neuroimaging meta-analytic platform with 30 578 functional MRI studies was used to derive meta-analytic task activation maps. We selected activation maps corresponding to 123 cognitive terms based on the Cognitive Atlas framework.^42^ Partial least-squares regression analysis was used to examine the spatial relationship between fused topological deviation maps and cognitive terms across biotypes (eMethods in Supplement 1).

#### Out-of-Sample Validation of Biotypes

To assess the generalizability of our ADHD clustering model, we transferred the pretrained model to an independent validation dataset from the Healthy Brain Network, which underwent identical processing steps as the discovery sample up to the normative model training. A transfer learning strategy was used to recalibrate the reference normative range model using TDC samples from the Healthy Brain Network as adaptation data to accommodate site-specific variations.^43^ We then generated estimates of the validation sample to characterize their topological deviations. By leveraging HYDRA algorithm parameters that defined a set of hyperplanes, we reconstructed the polytope to compute expression scores (Figure 1H and eMethods in Supplement 1). Individuals with ADHD in the validation cohort were then assigned to clusters based on their maximum expression scores, with examination on their clinical profiles and neural deviation patterns. Study data were analyzed from November 2023 to January 2025.

## Results

### Clinical Characteristics of Included Data

We included 446 children diagnosed with ADHD (mean [SD] age, 11.5 [2.7] years; 107 female [24%]; 339 male [76.0%]) and 708 TDCs (mean [SD] age, 11.0 [2.3] years; 279 female [39.4%]; 429 male [60.6%]) in the discovery cohort. We included 554 children with ADHD (mean [SD] age, 10.1 [2.8]; 182 female [32.9%]; 372 male [67.1%]) and 123 TDCs (mean [SD] age, 10.1 [3.0]; 53 female [43.1%]; 70 male [56.9%]) in the validation cohort (selection process detailed in eFigures 1 and 2 in Supplement 1). Demographic and phenotypical characteristics across sites, along with scan parameters, are provided in eTables 3 and 4 in Supplement 1, respectively. The study workflow is depicted in Figure 1A.

### Individual Deviations in Topological Properties

**Figure 2.**
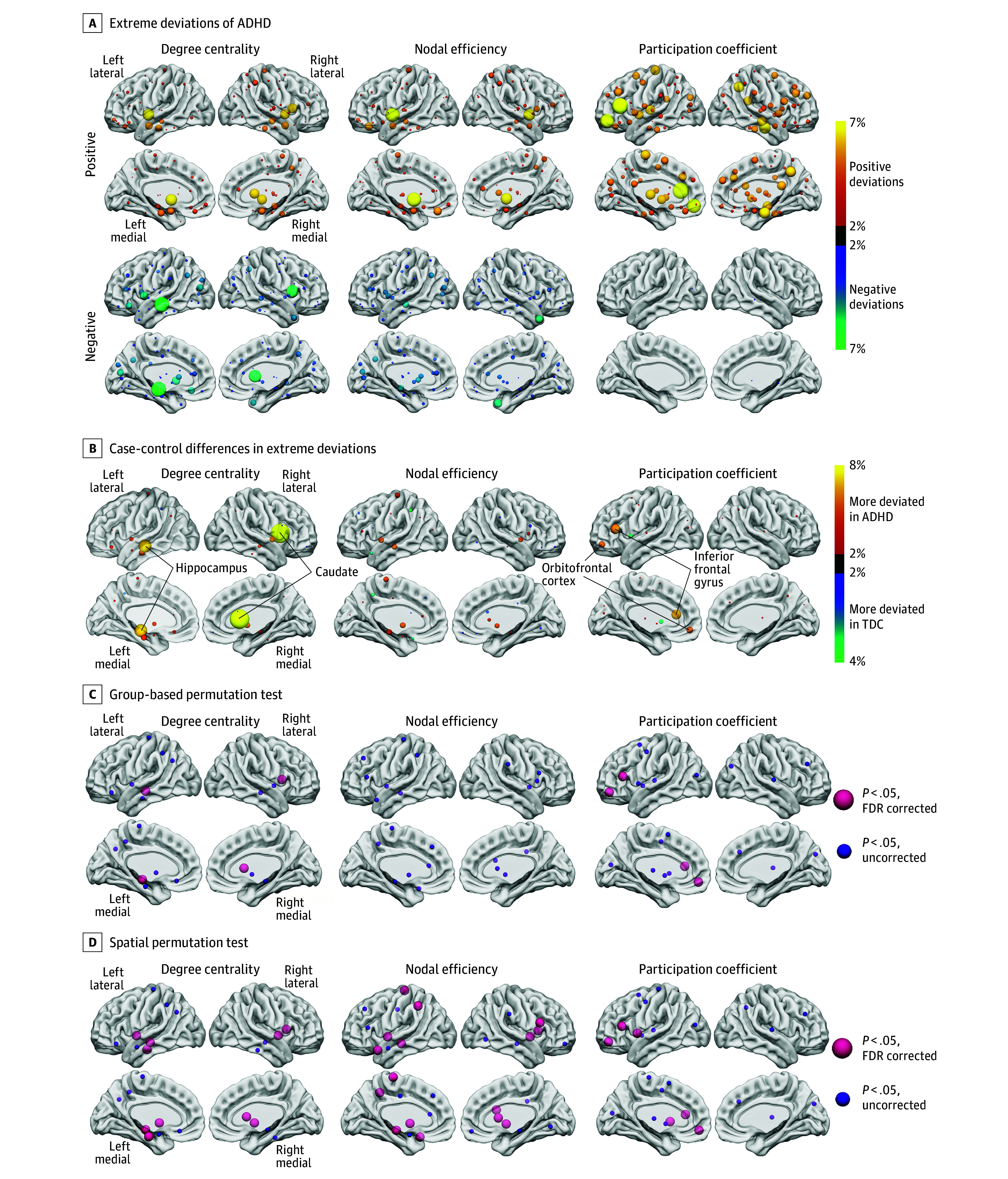
Nodal Heterogeneity of Extreme Topological Deviations A, Proportion of individuals displaying extreme topological deviations across brain nodes (brighter color indicates higher proportion). B, Differential patterns between groups (subtracting typically developing control overlap maps from attention-deficit/hyperactivity disorder [ADHD] overlap maps). Nodes that survived both group-based and spatial permutation tests are indicated with black solid lines. C and D, Nodes exhibiting significant case-control differences in extreme deviation patterns, assessed by both group-based and spatial permutation tests.

A multimetric approach that incorporated 3 topological metrics was used to assess each region’s MSN hubness.^38,44^ Fitting performance of our normative models was illustrated in eFigure 3 in Supplement 1 with examination of site effects presented in eTable 5 in Supplement 1. Among children with ADHD, 95.1% (424 of 446), 96.4% (430 of 446), and 71.8% (320 of 446) exhibited at least 1 extreme deviation in degree centrality, nodal efficiency, and participation coefficient, respectively, from the normative range. Compared with TDCs, children with ADHD showed significantly more extreme deviations per individual in degree centrality (mean [SD], 4.54 [2.96] vs 4.19 [2.82]; *t* = 2.05, Cohen *d* = 0.12; *P* = .04) and participation coefficient (mean [SD], 3.27 [4.22] vs 2.63 [3.53]; *t* = 2.78, Cohen *d* = 0.17; *P* < .001), but not nodal efficiency (mean [SD], 4.45 [2.79] vs 4.36 [2.69]; *t* = 0.57, Cohen *d* = 0.04; *P* = .57) (eFigure 4 in Supplement 1). The proportion of children with suprathreshold positive or negative deviations at each topological node revealed distinct patterns across topological metrics (Figure 2A).

Case-control comparison at the level of extreme deviation overlap (Figure 2B and C and eTable 6 in Supplement 1) revealed notable degree centrality differences in the caudate and hippocampus (observed difference = 7.79% and 6.33%; Cohen *h* = 0.30 and 0.24, respectively; FDR *P* <.001), and participation coefficient differences were primarily identified in the inferior frontal gyrus and orbitofrontal cortex (5.50% and 4.52%; Cohen *h* = 0.24 and 0.20, respectively; FDR *P* ≤.045). For nodal efficiency, significant spatial overlap patterns emerged in the hippocampus and pallidum (4.31% and 4.24%; Cohen *h* = 0.17 and 0.15, respectively; both FDR *P *<.001 in spatial permutation testing).

### Covarying Multimodal Signatures

**Figure 3.**
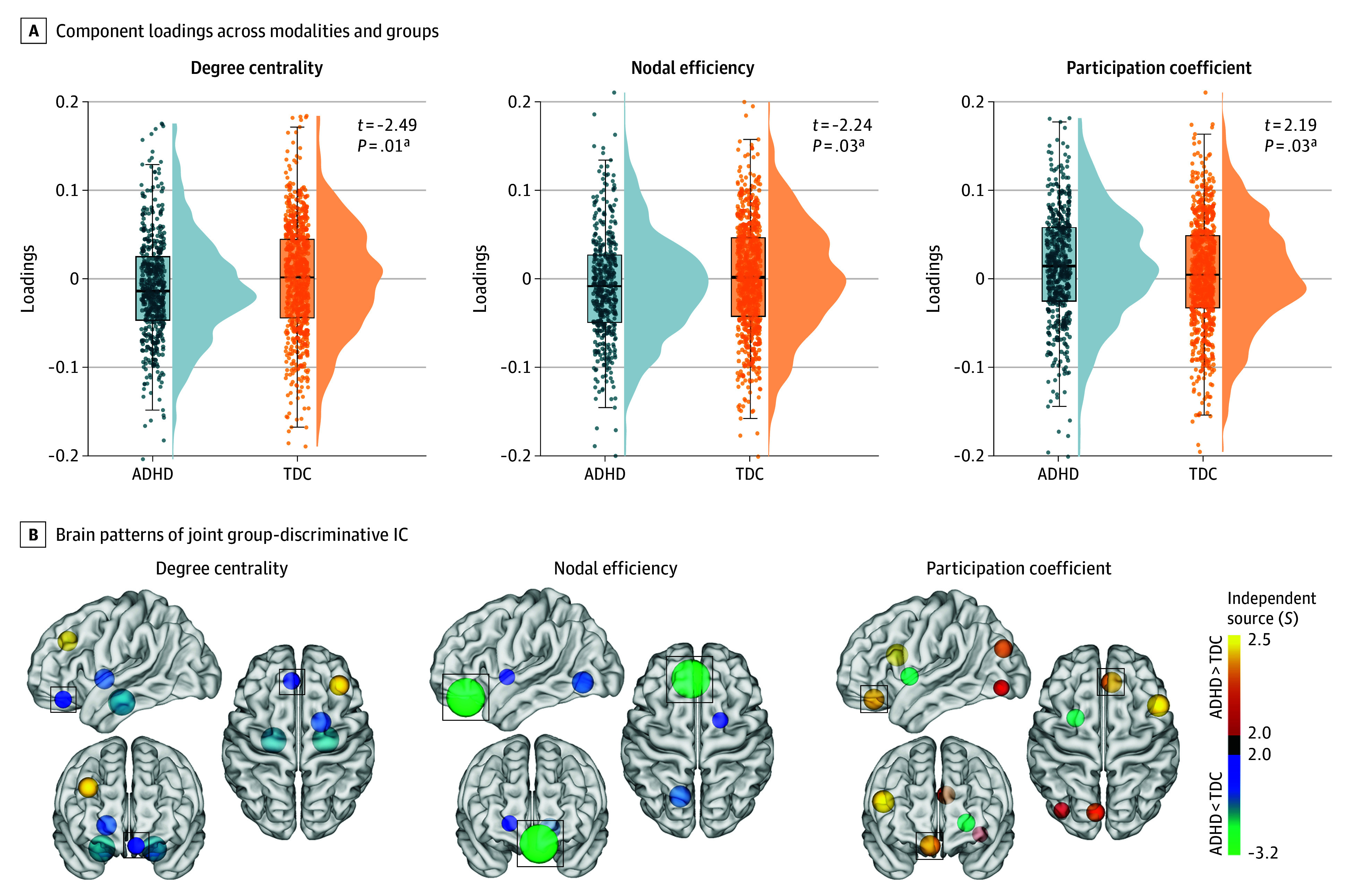
Case-Control Differences in Covarying Topological Signatures A, Component loadings across modalities and groups, with statistical comparisons. B, Spatial distribution of the independent component (IC) based on independent source (*S*). Consistent alterations across topological metrics were highlighted with black squares. ADHD indicates attention-deficit/hyperactivity disorder; TDC, typically developing control.

Having established individual deviation patterns across topological metrics, we applied mCCA + jICA to validate our multimetric approach and summarize topological findings. The optimal selection of 8 components preserved a substantial proportion of the explained variance across metrics (80.7%, 83.2%, and 82.9% for degree centrality, nodal efficiency, and participation coefficient, respectively). Notably, the fusion model yielded a joint component that demonstrated significant case-control differences in mixing coefficients across topological metrics (*t* = −2.49, Cohen *d* = −0.15; *P* = .01; *t* = −2.24, Cohen *d* = −0.14; *P* = .03; and *t* = 2.19, Cohen *d* = 0.13; *P* = .03, respectively) (Figure 3A). Within this group-differentiating component, covarying patterns were predominantly localized to the orbitofrontal cortex and were significant across topological metrics (*z* = −2.11, −3.15, and 2.31, respectively) (Figure 3B and eTable 7 in Supplement 1).

### ADHD Biotypes Corresponding to Clinical Profiles

The 3-biotype solution best captured the heterogeneity of topological deviations with high robustness and reliability according to 4 criteria: the highest adjusted Rand indices (0.21 for *k* = 3, 0.18 for *k* = 2, and 0.17 for *k* = 4); alternative adjusted Rand indices (*k* = 3) were significantly higher than null expectations; neural deviation patterns excluding females were similar compared with the full-sample model; *k* = 3 emerged as the optimal solution in split-half cross-validation based on adjusted Rand indices with the clustering scheme demonstrating replicability in neural patterns (eFigures 5-8 in Supplement 1). Cluster-wise margin distance and assignment entropy are available in eTable 8 in Supplement 1.

**Figure 4.**
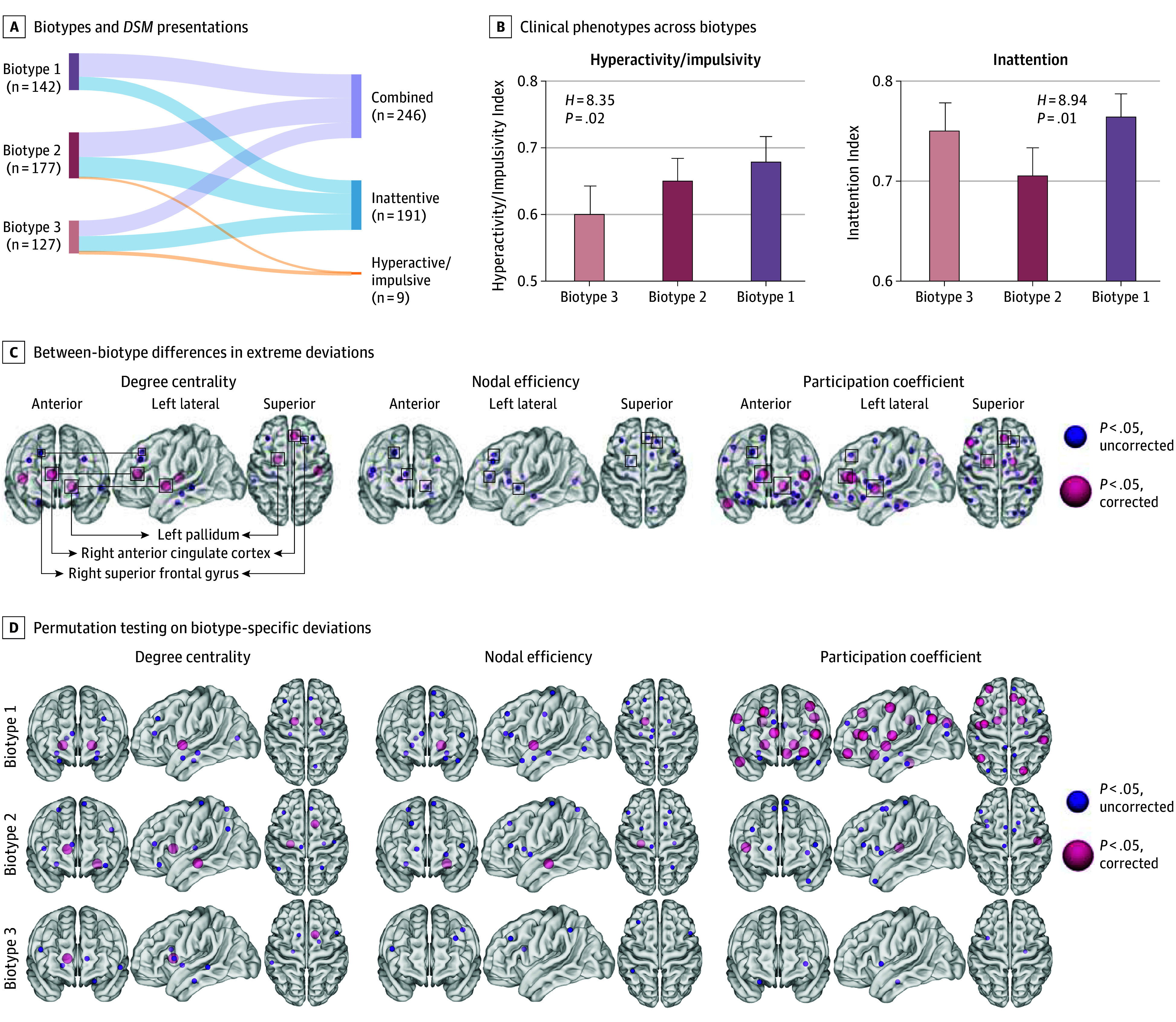
Attention-Deficit/Hyperactivity Disorder (ADHD) Biotype Identification Based on Heterogeneity Through Discriminative Analysis Modeling A, Correspondence between 3 identified ADHD biotypes and *DSM*-based presentations. The width of each flow represents the number of participants. B and C, Between-biotype differences in symptom severity and atypical neural mechanisms. Neuroanatomical locations of hub regions are shown, with smaller purple nodes indicating suprathreshold significance at uncorrected *P* <.05 and larger pink nodes indicating suprathreshold significance at false-discovery rate *P* <.05. Consistent alterations across topological properties were highlighted with squares. D, Hub nodes that exhibited statistical significance in group-based permutation tests when compared to null patterns. H indicates the Kruskal-Wallis test statistic.

Biotypes 1, 2, and 3 consisted of 142, 177, and 127 children with ADHD, respectively (relationship with *DSM* presentations in Figure 4A). They showed distinct clinical manifestations across putative biotypes, with significant differences in both inattention measured by Connors Rating Scale or ADHD Rating Scale (*H* = 8.94; *η^2^* = 0.016; *P* = .01) (Figure 4B) and hyperactivity/impulsivity (*H* = 8.35; *η^2^* = 0.014; *P* = .02). The biotype 1 (severe-combined with emotional dysregulation with widespread medial prefrontal cortex-pallidum alterations) exhibited the most elevated symptomatology, with the highest scores in both inattention (mean [SD], 0.77 [0.14]) and hyperactivity/impulsivity (mean [SD], 0.68 [0.22]). In contrast, biotype 2 (predominantly hyperactive/impulsive with anterior cingulate cortex-pallidum circuit alterations) and biotype 3 (predominantly inattentive with superior frontal gyrus alterations) showed distinct profiles with biotype 2 demonstrating higher hyperactivity/impulsivity (mean [SD], 0.65 [0.23] vs 0.60 [0.24]) but lower inattention (mean [SD], 0.71 [0.19] vs 0.75 [0.16]) compared with biotype 3. Post hoc comparisons identified significant differences in hyperactivity/impulsivity between biotypes 1 and 3 (*t* = 2.87, Cohen *d* = 0.35; adjusted *P* = .01) and inattention between biotypes 1 and 2 (*t* = 2.81, Cohen *d* = 0.34; adjusted *P* = .02).

When examining nodal between-biotype differences (Figure 4C), the right anterior cingulate cortex (χ^2 ^≥12.16; *P* ≤ .002), left pallidum (χ^2 ^≥11.33; *P* ≤ .003), and right superior frontal gyrus (χ^2 ^≥7.75; *P* ≤ .02) demonstrated consistent alterations across topological metrics (eTable 9 in Supplement 1 for modality-specific differences). Relative to others, biotype 1 exhibited the most extreme deviations across the above nodes and all metrics, particularly in participation coefficient (6.34%, 13.38%, and 9.15%, respectively). Biotype 2 instead demonstrated more atypical patterns of degree centrality in the left pallidum (7.34%) and nodal efficiency in the right anterior cingulate cortex (9.04%), whereas biotype 3 showed greater alterations in the right superior frontal gyrus (7.09% and 8.70% in degree centrality and nodal efficiency, respectively). Biotype 1 exhibited the most extensive deviations (45 significant deviated hub-metric combinations), whereas biotypes 2 and 3 showed 26 and 11 deviations compared with normative ranges (Figure 4D and eFigure 9 in Supplement 1, with detailed comparisons in eTables 10 and 11 in Supplement 1).

Analysis of longitudinal trajectories in medication-naive individuals with ADHD revealed no significant time × biotype interaction effects (eTable 12 in Supplement 1) for both attention and externalizing problems. Intriguingly, when examining deficient emotional self-regulation, biotype 1 showed more persistent symptoms compared to the marked decreases observed in biotypes 2 and 3 (biotype 1 vs 2: *z* = −2.11, Cohen *d* = −0.24; *P* = .04; biotype 1 vs 3: *z* = −2.66, Cohen *d* = −0.33; *P* = .008) (eFigure 10 in Supplement 1). Regarding the development of mood disorders, although biotype 1 showed a higher rate of mood disorder comorbidity (25.0%) compared with biotypes 2 and 3 (9.8% and 5.0%, respectively), although without statistical significance (*χ^2^* = 2.89; *P* = .22). Medication usage patterns across biotypes in follow-up samples were provided in eTable 13 in Supplement 1, without significant differences observed.

### Biotype Decoding

**Figure 5.**
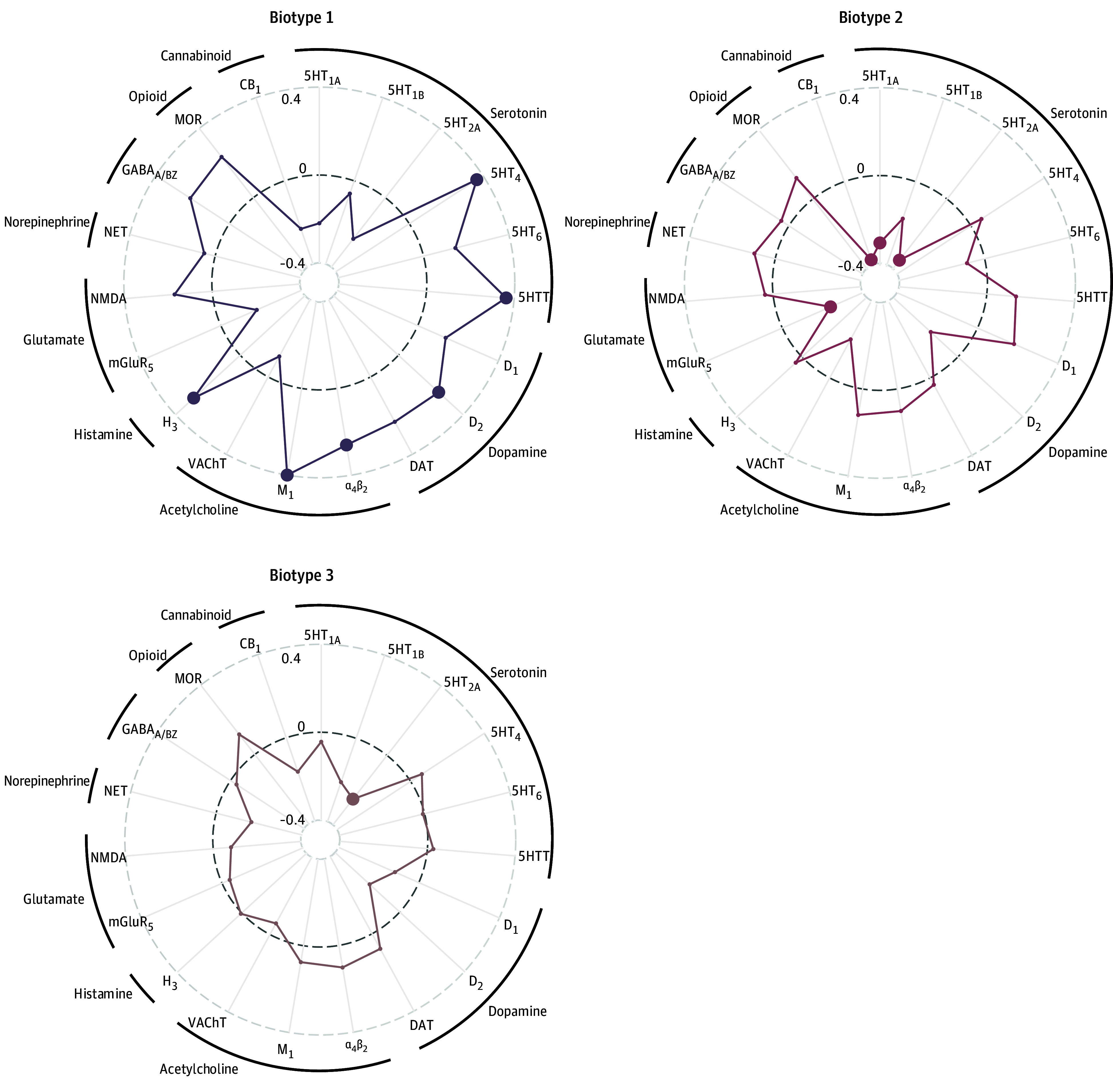
Spatial Correlations of Neurotransmitters With Biotype-Specific Deviations Spatial correlations between maps of fused topological deviations and neurotransmitter receptor density distributions across biotypes. Each axis represents a distinct receptor subtype, with outer circles indicating broader neurotransmitter systems. Distance from the dashed baseline reflects correlation strength. Solid dots indicate statistically significant correlations after correction for spatial autocorrelation using spin tests (spin *P* <.05). CB_1_ indicates cannabinoid receptor 1; DAT, dopamine transporter; D_1_, dopaminergic receptor 1; D_2_, dopaminergic receptor 2; GABA, γ-aminobutyric acid; H_3_, histaminergic receptor 3; 5HT, 5-hydroxytryptamine receptor; 5HTT, 5-hydroxytryptamine transporter; mGluR_5_, metabotropic glutamate receptor 5; MOR, μ-opioid receptor; NET, norepinephrine transporter; NMDA, *N*-methyl-d-aspartate; VAChT, vesicular acetylcholine transporter.

Regarding the molecular bases of our topology-derived ADHD biotypes, spatial relationships with 19 neurotransmitter density maps were drawn (eFigure 11 in Supplement 1), revealed that topological abnormalities in biotype 1 of ADHD exhibited significant spatial correspondence with the serotonin (5-hydroxytryptamine receptor 4 [5-HT_4_], Pearson *r* = 0.37, spin-FDR *P* = .003; 5-hydroxytryptamine transporter [5-HTT], Pearson *r* = 0.37, spin-FDR *P* = .005), dopamine (dopaminergic receptor 2 [D_2_], Pearson *r* = 0.25, spin-FDR *P* = .03), acetylcholine (α_4_β_2_, Pearson *r* = 0.26, spin-FDR *P* = .03; muscarinic receptor 1 [M_1_], Pearson *r* = 0.41, spin-FDR *P* <.001), and histamine (histaminergic receptor 3 [H_3_], Pearson *r* = 0.29, spin-FDR *P* = .02) neurotransmitter density distributions (Figure 5). The atypical patterns of biotype 2 showed significant anticorrelations with glutamate (metabotropic glutamate receptor [mGluR_5_], Pearson *r* = −0.24, spin-FDR *P* = .03), cannabinoid (CB_1_, Pearson *r* = −0.37, spin-FDR *P* <.001), and serotonin (5-HT_1A_, Pearson *r* = −0.30, spin-FDR *P* <.001; 5-HT_2A_, Pearson *r* = −0.34, spin-FDR *P* <.001) systems, whereas biotype 3 selectively exhibited significant anticorrelations with a serotonin receptor (5-HT_2A_, Pearson *r* = −0.25, spin-FDR *P* = .03).

To validate the ADHD biotypes’ clinical profiles and broaden our understanding beyond the checklists, we examined associations between each biotype and Neurosynth-derived activation maps of psychological processes.^40^ We identified partial least-squares components with highest explained variance across maps of cognitive terms, accounting for 12.7%, 9.6%, and 8.5% of variance for biotypes 1 to 3, respectively (spin *P* = .01, spin *P =*.04, and spin *P* =.048, respectively). The contribution of cognitive terms to partial least-squares components across biotypes was hierarchically organized in bootstrapping (eFigure 12 and eTable 14 in Supplement 1). Three biotypes exhibited distinct cognitive profiles: biotype 3 was characterized by predominant attention compared with impulsivity + hyperactivity (|Z| = 4.09 vs 0.40 + 0.94), whereas biotype 2 showed an inverse pattern (|Z| = 1.48 vs 2.48 + 0.47), and biotype 1 demonstrated comparable expression across domains (|Z| = 1.52 vs 3.34 + 2.89).

### Biotype Validation

In validating our model’s generalizability, the pretrained HYDRA clustering solution identified 3 ADHD biotypes in the validation cohort, with 173, 204, and 177 children assigned to biotypes 1 to 3, respectively. Significant differences in the hyperactive/impulsive domain between biotypes (assessed by Conners Rating Scale, *H* = 6.58; *η^2^* = 0.011; *P* = .04) maintained consistency with the discovery cohort, showing a decreasing severity profile from biotype 1 to 3 (eFigure 13A in Supplement 1). Post hoc comparisons also demonstrated significant differences between biotypes 1 and 3 (mean [SD], 0.71 [0.14] vs 0.67 [0.14]; *t* = 2.55; adjusted *P = *.03). However, the inattention profiles showed no significant between-biotype differences (*H* = 4.14; *η^2^* = 0.005; *P* = .13). We also used the Child Behavior Checklist to validate our findings, showing that distinct patterns of hyperactivity/impulsivity (*H* = 7.19; *η^2^* = 0.009; *P* = .03; between biotypes 1 and 3: mean [SD], 0.66 [0.12] vs 0.63 [0.13]; *t* = 2.48; adjusted *P* = .04). Patterns of attention problems in the validation cohort paralleled those of the discovery sample without statistical significance (*H* = 0.49; *η^2^* = −0.003; *P* = .78).

Biotype-specific topological deviation patterns showed significant consistency between training and validation cohorts, evidenced by high correlations between their averaged feature vectors (biotype 1, Pearson *r* = 0.72; biotype 2, Pearson *r* = 0.80; biotype 3, Pearson *r* = 0.76; all *P* < .001). The strongest between-biotype differences emerged with respect to participation coefficient deviations (16 significant areas), paralleling observations from the discovery cohort (eFigure 13B and eTable 15 in Supplement 1). Biotype-specific neural bases in the validation cohort are shown in eFigure 13C and D and eTable 16 in Supplement 1). Nodes demonstrating consistent extreme deviations across both discovery and validation cohorts are presented in eFigure 14 in Supplement 1.

## Discussion

Phenotypic heterogeneity poses a major challenge in the diagnosis and treatment of ADHD,^5^ as the current diagnostic framework assigns a single diagnostic label to what is fundamentally a heterogeneous syndrome that likely arises from diverse neural mechanisms.^33^ Our study advances the understanding of ADHD heterogeneity through a novel hub-oriented fusion framework that integrates multimodal topological deviations in morphometric similarity networks. The replication of our findings across 2 cohorts suggests that our approach may offer a feasible framework for stratifying ADHD cases. Overall, our integrated approach adheres to the recommended workflows for investigating ADHD heterogeneity.^4,45^

### Brain-First Approach for ADHD Subtyping

This brain-first approach provides biological validation of identified subtypes through completely data-driven clustering. Although *DSM* classifications exclusively rely on consensus-derived symptoms, our neuroimaging-derived clusters converged with clinical phenotypes without using any clinical features. This convergence provides compelling evidence that these presentations reflect genuine neurobiological entities, biologically validating these long-observed clinical distinctions. Although partial overlap emerges in the distributions of topologically deviated hubs between biotypes, our multimetric topological approach enables comprehensive characterization of subtle network-level deviations that may be obscured by single-metric or symptom-based analyses.^4^ Robust classification boundaries could be identified within multivariate neuroimaging landscape for subgroup differentiation, remaining clinically valuable for adjudicating distinct management strategies.^2^ These circuit-specific mechanisms may provide potential targets for stratified interventions tailored to biotype’s unique network dysfunction profile. Critically, biotype 1, marked by the most widespread deviation patterns, reveals a clinically valuable subgroup with distinct developmental trajectories requiring early interventions, providing neurobiological markers of vulnerability.^46,47^

### Topological Deviations of ADHD From Normative Centiles

Our finding that children diagnosed with ADHD exhibit prominent degree centrality and nodal efficiency deviations in subcortical hubs, particularly the striatum, suggests a weakened functionality consistent with Enhancing Neuroimaging Genetics through Meta-analysis (ENIGMA)–ADHD consortium findings of reduced subcortical gray matter volume.^48^ Given the altered participation coefficient patterns, the roles of inferior frontal gyrus and orbitofrontal cortex may shift from provincial hubs to connector hubs in ADHD,^49^ representing compensatory adaptation to reduced processing capacity in other nodes, manifesting as early increases.^50^ The classical ADHD model of pathophysiology suggests delayed brain maturation,^51^ particularly in frontal cortex and frontostriatal connections.^52^ Prefrontal areas, particularly the inferior frontal gyrus,^53^ and their associated networks could act as a brake for inhibitory control and decision-making optimization, and striatal inhibitory projections that modulate dopaminergic neuron activity may drive impulsivity.^54,55,56^

### Characterizations of Distinct Biotypes

Our multimodal fusion analysis identified the orbitofrontal cortex as a common covarying component across topological metrics, suggesting it may serve as a fundamental network anchor point in ADHD pathophysiology that transcends biotype boundaries. This finding aligns with the orbitofrontal cortex’s role of orchestrating the transition between impulsive and reflective behaviors engaged with goal-directed actions.^57,58^ Building on this shared foundation, biotype-specific deviations emerge in distinct neural circuits: widespread medial prefrontal cortex-pallidum alterations in biotype 1 (severe-combined with emotional dysregulation) could indicate dysregulation in frontostriatal circuitry^59^; anterior cingulate cortex-pallidum circuit alterations in biotype 2 (predominantly hyperactive-impulsive) may preferentially drive hyperactive/impulsive behaviors through dysregulated action-mode networks^60^; and superior frontal gyrus alterations in biotype 3 (predominantly inattentive) may selectively impair sustained attention through default mode network interference^2^ (a discussion about parallel and distinct neural dysfunctions is available in the eDiscussion in Supplement 1). This hierarchical model, in which orbitofrontal cortex dysfunction represents a common substrate while region-specific deviations shape individual-specific symptomatic profiles, may explain why certain neural signatures appear consistently in case-control comparisons, whereas others emerge only through biotype stratification.

### Neurochemical Contextualization Beyond Biotypes

The 3 biotypes exhibited distinct spatial correspondences with neurotransmitter receptor distributions as exploratory contextualization. Past work has documented roles of these neurotransmitter systems in ADHD, including dopaminergic-serotonergic interactions through orbitofronto-striatal circuits and cannabinoid system alterations in hyperactivity/impulsivity^61,62,63^; cholinergic dysfunction affecting motor control and attention in combined presentations^64,65^; histaminergic involvement in neuroinflammatory pathophysiology^66^; glutamate alterations in reward-processing caudate circuits^67^; and serotonergic contributions to emotional dysregulation.^68^ Our analysis provides preliminary evidence that these neurotransmitter systems may be differentially involved across biotypes,^36,69^ but they cannot represent actual alterations in receptor systems themselves or inform treatment approaches.

### Cognitive Validation and External Replication

Our Neurosynth-decoding findings demonstrated robust alignment between biotype-dominant clinical profiles and expression patterns of their corresponding cognitive terms. This correspondence to a large-scale database validates our ADHD biotype classification system derived from topological deviations. Further, the application of a pretrained model in the validation cohort substantially externally replicated the clustering patterns observed in the discovery datasets. Specifically, the validation cohort exhibited consistent hyperactive/impulsive patterns across biotypes, although inattention profiles could not be fully reproduced, likely due to confounding factors including younger participant age, different recruitment strategies, and cross-cultural variations.

### Limitations

Several limitations should be considered. First, our samples of participants were not medication naive. Although psychostimulant use does not significantly influence brain morphometric findings in ADHD mega-analysis,^48,70^ we cannot rule out their potential confounding effects. Our exclusion criteria for comorbid conditions may limit our generalizability to real-world clinical settings where comorbidities are prevalent. Second, the overlap in topological deviations between ADHD and TDCs underscores the subtle and complex nature of ADHD-related brain alterations. This complexity may reflect the inherent challenges in identifying discrete neurobiological markers.^27^ As morphology-derived correlation matrices, network configurations of MSNs require cautious mechanistic interpretation.^71,72,73^ Third, our putative biotypes may represent salient points along underlying dimensional continua rather than qualitatively distinct diagnostic entities. Future directions for clinical translation were discussed in the eDiscussion in Supplement 1.

## Conclusions

In this case-control study, we have identified 3 distinct topology-derived biotypes, each characterized by unique clinical-neural profiles, longitudinal trajectories, and spatial molecular signatures, with rigorous validation through cognitive profiles from large-scale databases and independent cohorts. Our comprehensive approach, from feature extraction through data-driven clustering to external validation, offers a promising framework for parsing the inherent ADHD heterogeneity in a clinically valuable way, which may ultimately create a path toward developing personalized therapeutic strategies.

## Notes

Supplement 1.
eMethods.

eDiscussion.
**eFigure 1.** Sample Selection Flowchart for Discovery Cohort **eFigure 2.** Sample Selection Flowchart for Validation Cohort **eFigure 3.** Performance Metrics Across Nodes for Normative Models **eFigure 4.** Number of Extreme Deviations Across Groups **eFigure 5.** Permutation Testing for Statistical Significance of Clustering **eFigure 6.** Extreme Deviation Patterns Among Biotypes in Boys **eFigure 7.** Adjusted Rand Indices in Split-Half Cross-Validation **eFigure 8.** Stratified Split-Half Cross-Validation for Clustering **eFigure 9.** Biotype-Specific Extreme Deviation Patterns **eFigure 10.** Biotype-Specific Longitudinal Trajectories of Deficient Emotional Self-Regulation **eFigure 11.** Fused Topological Deviations Across Biotypes **eFigure 12.** Biotype-Specific Functional Correlates **eFigure 13.** Biotype Validation Based on Pretrained HYDRA Mode **eFigure 14.** Cross-Cohort Consistency of Hub Node Alterations **eTable 1.** Inclusion and Exclusion Criteria Across Sites in Discovery Cohort **eTable 2.** Included PET Studies on Neurotransmitter Distribution **eTable 3.** Demographic and Phenotypical Measures of Datasets Across Sites **eTable 4.** Scan Parameters of Datasets Across Sites **eTable 5.** Cross-Site Validation of Observed Topological Deviations **eTable 6.** Nodal Metrics With Significant Case-Control Differences **eTable 7.** Brain Patterns of Joint Component of Topological Metrics **eTable 8.** Cluster-Wise Margin Distance and Assignment Entropy **eTable 9.** Between-Biotype Differences Across Topological Modalities **eTable 10.** Comparison of Number of Extreme Deviations Across Biotypes **eTable 11.** Biotype-Specific Extreme Deviation Patterns **eTable 12.** Linear Mixed-Effect Models for Longitudinal Changes **eTable 13.** Medication Usage Across Biotypes in Follow-Up Samples **eTable 14.** Contribution of Cognitive Terms to PLS1 Components **eTable 15.** Between-Biotype Differences in Validation Cohort **eTable 16.** Biotypes-Specific Extreme Deviation Patterns in Validation Sample

Supplement 2.
Data Sharing Statement.
