# Supplementary material for: Mapping ADHD Heterogeneity and Biotypes by Topological Deviations in Morphometric Similarity Networks
Source: JAMA Psychiatry. 2026 Feb 25;83(5):478–90. doi: 10.1001/jamapsychiatry.2026.0001 (PMC12936971; doi:10.1001/jamapsychiatry.2026.0001)
Supplement: Supplement 1. — eMethods. eDiscussion. eFigure 1. Sample Selection Flowchart for Discovery Cohort eFigure 2. Sample Selection Flowchart for Validation Cohort eFigure 3. Performance Metrics Across Nodes for Normative Models eFigure 4. Number of Extreme Deviations Across Groups eFigure 5. Permutation Testing for Statistical Significance of Clustering eFigure 6. Extreme Deviation Patterns Among Biotypes in Boys eFigure 7. Adjusted Rand Indices in Split-Half Cross-Validation eFigure 8. Stratified Split-Half Cross-Validation for Clustering eFigure 9. Biotype-Specific Extreme Deviation Patterns eFigure 10. Biotype-Specific Longitudinal Trajectories of Deficient Emotional Self-Regulation eFigure 11. Fused Topological Deviations Across Biotypes eFigure 12. Biotype-Specific Functional Correlates eFigure 13. Biotype Validation Based on Pretrained HYDRA Mode eFigure 14. Cross-Cohort Consistency of Hub Node Alterations eTable 1. Inclusion and Exclusion Criteria Across Sites in Discovery Cohort eTable 2. Included PET Studies on Neurotransmitter Distribution eTable 3. Demographic and Phenotypical Measures of Datasets Across Sites eTable 4. Scan Parameters of Datasets Across Sites eTable 5. Cross-Site Validation of Observed Topological Deviations eTable 6. Nodal Metrics With Significant Case-Control Differences eTable 7. Brain Patterns of Joint Component of Topological Metrics eTable 8. Cluster-Wise Margin Distance and Assignment Entropy eTable 9. Between-Biotype Differences Across Topological Modalities eTable 10. Comparison of Number of Extreme Deviations Across Biotypes eTable 11. Biotype-Specific Extreme Deviation Patterns eTable 12. Linear Mixed-Effect Models for Longitudinal Changes eTable 13. Medication Usage Across Biotypes in Follow-Up Samples eTable 14. Contribution of Cognitive Terms to PLS1 Components eTable 15. Between-Biotype Differences in Validation Cohort eTable 16. Biotypes-Specific Extreme Deviation Patterns in Validation Sample [file jamapsychiatry-e260001-s001.pdf]

Supplementary Online Content

Pan N, Long Y, Qin K, et al. Mapping ADHD heterogeneity and biotypes by topological deviations in morphometric similarity networks. *JAMA Psychiatry*. Published online February 25, 2026. doi:10.1001/jamapsychiatry.2026.0001

eMethods.

eDiscussion.

eFigure 1. Sample Selection Flowchart for Discovery Cohort

eFigure 2. Sample Selection Flowchart for Validation Cohort

eFigure 3. Performance Metrics Across Nodes for Normative Models

eFigure 4. Number of Extreme Deviations Across Groups

eFigure 5. Permutation Testing for Statistical Significance of Clustering

eFigure 6. Extreme Deviation Patterns Among Biotypes in Boys

eFigure 7. Adjusted Rand Indices in Split-Half Cross-Validation

eFigure 8. Stratified Split-Half Cross-Validation for Clustering

eFigure 9. Biotype-Specific Extreme Deviation Patterns

eFigure 10. Biotype-Specific Longitudinal Trajectories of Deficient Emotional Self-Regulation

eFigure 11. Fused Topological Deviations Across Biotypes

eFigure 12. Biotype-Specific Functional Correlates

eFigure 13. Biotype Validation Based on Pretrained HYDRA Mode

eFigure 14. Cross-Cohort Consistency of Hub Node Alterations

eTable 1. Inclusion and Exclusion Criteria Across Sites in Discovery Cohort

eTable 2. Included PET Studies on Neurotransmitter Distribution

eTable 3. Demographic and Phenotypical Measures of Datasets Across Sites

eTable 4. Scan Parameters of Datasets Across Sites

© 2026 Pan N et al. *JAMA Psychiatry*.

eTable 5. Cross-Site Validation of Observed Topological Deviations

eTable 6. Nodal Metrics With Significant Case-Control Differences

eTable 7. Brain Patterns of Joint Component of Topological Metrics

eTable 8. Cluster-Wise Margin Distance and Assignment Entropy

eTable 9. Between-Biotype Differences Across Topological Modalities

eTable 10. Comparison of Number of Extreme Deviations Across Biotypes

eTable 11. Biotype-Specific Extreme Deviation Patterns

eTable 12. Linear Mixed-Effect Models for Longitudinal Changes

eTable 13. Medication Usage Across Biotypes in Follow-Up Samples

eTable 14. Contribution of Cognitive Terms to PLS1 Components

eTable 15. Between-Biotype Differences in Validation Cohort

eTable 16. Biotypes-Specific Extreme Deviation Patterns in Validation Sample

This supplementary material has been provided by the authors to give readers additional information about their work.

© 2026 Pan N et al. *JAMA Psychiatry.*

eMethods

1. Processing for Structural Images

To perform retrospective image quality checks, the Computational Anatomy Toolbox (CAT12) was used to automatically identify problems in each T1-weighted image and provide a weighted overall image quality rating (IQR, IQR<3.5 for satisfactory quality)[1].

During voxel-based morphometry analysis, individual images were segmented into probability maps of gray matter, white matter, and cerebrospinal fluid tissue, then spatially normalized to the Montreal Neurological Institute (MNI) standard space with consistent 1.5mm isotropic voxels through the Diffeomorphic Anatomical Registration Exponentiated Lie Algebra (DARTEL) pipeline[2]. The modulation procedure implicitly accounted for total intracranial volume by preserving volumetric information while controlling for individual brain size differences. Voxel-wise GMV maps were then produced following bias-field correction and modulation using both linear and nonlinear components of the Jacobian determinant[3], and we employed spatial smoothing with 8mm full-width at half-maximum Gaussian kernel to improve the signal to noise ratio of GMV estimates.

The Automated Anatomical Labeling atlas was adopted to parcel the whole brain gray matter into 90 regions[4]. The probabilistic relationships based on Kullback-Leibler divergence similarity (KLS) between pairs were derived through kernel density estimation, which generated probability density functions for subsequent KLS computation. To optimize computational efficiency while maintaining statistical reliability, we implemented a sampling framework comprising 2^7 points[5].

2. Evaluated Graph Theoretic Metrics

Using the GRETNA toolbox[6], we applied a sparsity range of 0.10 to 0.34 (increments=0.01) to generate sparse networks with minimal spurious edges aligning with a small-world regime[7] and estimated the area under the curve to characterize the connectome independent of arbitrary sparsity thresholds[8].

(1) Measures of centrality

Degree centrality: The number of links connected to a node, which is also equal to the number of neighbors of a node. Nodes with a high degree are interacting structurally or functionally with many other nodes and might reflect their critical role in coordinating and integrating information across various cognitive processes [9].

© 2026 Pan N et al. *JAMA Psychiatry.*

$$DC_i = \sum_{j \in n} a_{ij}$$

Where a_{ij} is the connection status [a_{ij}=1 when link(i,j) exists, otherwise a_{ij}=0] between node i and node j, n is the set of all nodes in the network.

(2) Measures of integration

Nodal efficiency: The average inverse shortest path length, which could also be simplified as efficient connectivity between the two nodes, measuring the capacity for parallel information transfer and integrated processing among distributed components of the system. Higher global efficiency values suggest that the brain network is more interconnected and facilitates rapid communication between different brain regions [9].

$$NE_i = \frac{\sum_{j \in n, j \neq i} d_{ij}^{-1}}{n-1}$$

Where d_{ij} is the shortest path length between node i and node j.

(3) Measures of segregation

Participation coefficient: The diversity of intermodular connections of individual nodes, measuring the distribution of a node's edges among the communities. If the node's edges are evenly distributed among all communities, its participation coefficient could have the maximal value. Nodes with low participation coefficients are called provincial hubs because their edges are not distributed widely among communities, whereas hubs with higher participation coefficients are called connector hubs. We utilized a greedy optimization algorithm to identify the modular community appeared in the brain network [10]. Compared to pre-defined community partition, this algorithm starts with a situation in where every node in the network belongs to a sole module and ends up when the agglomeration no longer contributes to the optimization of the modularity, eventually accounting for the heterogeneity of modular size.

$$PC_i = 1 - \sum_{m=1}^{M} \left(\frac{k_{im}}{k_i}\right)^2$$

Where M is the total number of modular communities, k_{im} is the number of edges linking node i to other nodes that belong to the module m, k_i is the total number of linked edges of node i.

3. Basic Principles of Normative Modeling and its Settings

© 2026 Pan N et al. *JAMA Psychiatry.*

We employed warped Bayesian linear regression as our normative modeling algorithm based on recent evidence demonstrating its superior performance in modeling uncertainty and accommodating site effects[11]. Comparative studies have shown that it performs comparably to top-performing algorithms while effectively handling site effects, making it particularly suitable for multisite neuroimaging studies[12]. Rather than requiring explicit harmonization, normative modeling is more appropriate for quantifying individual deviations from normative references[13].

Prior to model fitting, we excluded subjects with non-fitting outliers ($|Z|>7$) and brain phenotypes were subjected to Yeo-Johnson transformations to mitigate skewness[3]. Our datasets were then strategically partitioned: 90% of typically developing controls (TDC) subjects from each site were allocated to the training dataset, while the remaining 10% of TDCs, together with all ADHD cases, constituted the test dataset. Including an independent subset of TDCs in the test set establishes a benchmark for deviations observed under normative conditions, allowing us to determine whether ADHD individuals show more extreme deviation patterns than expected under typical conditions[3,14]. Lastly, Bayesian linear regression was employed to estimate region-specific normative variance between covariates and deviations for each topological metric, and to model uncertainty. Specially, we utilized B-spline basis expansion (cubic spline with 5 evenly spaced knots) to model nonlinear effects of age in our normative models, and a bijective nonlinear warping function ('sinarcsinsh', extensively used in generalized additive modeling) was applied to transform non-Gaussian response variables into a Gaussian latent space, enabling closed-form inference while simultaneously accommodating both non-Gaussianity and nonlinear relationships in the data [15]. Site-related confounding effects were incorporated in the model [16]. Powell algorithm was used to optimize hyperparameters. The regression model equation of normative modeling is shown below [17]:

$$\mathrm{Y} = f(\mathrm{X}, \theta) + \mathrm{E}$$

Where Y is brain responses, X is covariates, θ is model parameters, and E is residuals.

To assess how much each subject deviates from the normative range, deviation maps (Z-score) were calculated based on the following formula [15]:

$$Z_{nd} = \frac{y_{nd} - \hat{y}_{nd}}{\sqrt{\sigma_d^2 + (\sigma_*^2)_d}}$$

© 2026 Pan N et al. *JAMA Psychiatry.*

For each subject n and each brain parcel d, where y_{nd} is the observed response, $\hat{y}_{nd}$ is the predicted mean, σ_d^2 is the predictive noise variance, and $(\sigma_*^2)_d$ is the normative variance. For TDCs, corresponding deviation patterns were established using 10-fold cross-validation.

4. Permutation Testing on Extreme Deviations

In the group-based permutation test [3], for both the ADHD and TDC groups, we quantified the prevalence of extreme deviations within each node. This was accomplished by counting subjects exhibiting extreme deviations ($Z \geq |2.0|$), considering both positive and negative extremes in each node. To ascertain the observed difference for individual nodes, we subtracted the surrogate healthy control overlap map from its clinical group map. Subsequently, we conducted a permutation analysis comprising 5,000 iterations. Each iteration involved the following steps: (1) randomization of group labels, (2) recalculation of overlap maps using the randomized labels, and (3) computation of the difference between the shuffled clinical and control overlap maps. This procedure generated a null distribution of difference maps under the hypothesis of random group assignment. For each node, we derived the p-value by calculating the proportion of permutation differences that were greater than or equal to the absolute value of the observed difference. Finally, we identified statistically significant differences using a threshold of $p_{FDR}<.05$ (two-tailed).

In the spatial permutation test, we generated an ensemble of 5,000 surrogate deviation maps for each participant, with BrainSMASH considering spatially autocorrelated measures [18]. These surrogate maps were thresholded at $Z \geq |2.0|$ to create surrogate extreme deviation maps, which were subsequently stacked. For both ADHD and TDC groups, we computed surrogate within-group overlap maps, by quantifying the proportion of individuals exhibiting a surrogate deviation within each node. We then calculated the difference between the clinical group's surrogate overlap map and the TDC surrogate overlap map to obtain a surrogate overlap difference map for each iteration. The *p*-value for each node was derived by determining the proportion of permutation differences that met or exceeded the absolute value of the observed difference. Finally, we identified statistically significant differences using a threshold of $p_{FDR}<.05$ (two-tailed).

5. Mathematic Principles and Settings of Multimodal Fusion

Briefly, mCCA identified inter-modal relationships by optimizing correlations among loading parameters (X: topological vectors, Y: derived canonical variates), then jICA delineated spatial maps to elucidate case-control variations.

© 2026 Pan N et al. *JAMA Psychiatry.*

The joint decomposition yielded individual-specific mixing coefficients (A_k, k=1,2,3) and independent sources (S_k) for each modality, and we normalized S_k and then thresholded at $|Z|\geq 2.0$ to visualize spatial maps.

Since we have three topological modalities (k=1,2,3), the topological features X_1 were assigned to degree centrality, X_2 to nodal efficiency, and X_3 to participation coefficient. For each modality k:

$$X_k = D_k C_k \text{ (mCCA)}$$

$$X_k = A_k S_k,\ C_k = S_k W^{-1} \text{ (jICA)}$$

During the mCCA processing, X_k were projected to generate two canonical variates (D_k and C_k) in a space that the correlations among mixing profiles D_k are jointly maximized in their sum of squared correlation, and derived associated maps C_k were linked to become concatenated[19]. Given that X_k is a linear mixture of M sources (minimum description length criterion was used to determine the optimal number of components[20], M=8 ICs in our study) given by S_k with a non-singular mixing coefficients A_k, jICA was then adopted on the concatenated maps C_k to obtain the maximally independent sources S_k and corresponding whitening matrix W[21].

To assess clinical relevance, we compared mixing coefficients between groups using two-sample *t* tests to identify joint group-differentiating component.

6. HYDRA Settings and Reproducibility Analyses

The individual deviation maps (i.e., Z scores of each brain regions) of three topological properties were inputted as features and age, sex, and site were entered as covariates in HYDRA modeling. HYDRA algorithms were conducted with the following default settings: 10-fold nested cross-validation, regularization parameter = 0.25, L1 regularization, assignment by random hyperplanes of determinantal point processes, 50 iterations between estimating hyperplanes and cluster estimation, and 25 clustering consensus steps[22]. Clustering stability was assessed using adjusted Rand indices (ARIs) to quantify reproducibility across cross-validation folds. The algorithm incorporates multiple stopping mechanisms: (a) Cross-validation stability - declining ARI scores with increasing k indicate over-subdivision in the nested cross-validation; (b) Consensus validation - the 25 consensus steps ensure only reproducible subtypes emerge, filtering out unstable subdivisions; and (c) Convergence criteria - iterative optimization (50 iterations) stops when cluster assignment changes fall below 1e-6, preventing arbitrary assignments. Specifically, the analysis was constrained to k = 2 to 4, as higher dimensionality was deemed impractical in clinical settings of ADHD.

© 2026 Pan N et al. *JAMA Psychiatry.*

The robustness of the identified ADHD biotypes was validated via three reproducibility analyses. First, we conducted permutation testing to generate null distributions and compared their corresponding ARIs with those derived from a null model generated by randomly assigning TDC subjects into pseudo-groups to determine the statistical significance for the optimal *k*-cluster solution[22]. Second, since ADHD is more commonly diagnosed in boys, we also repeated our clustering analysis after excluding female participants, to account for any sex-specific effects. Finally, we executed a split-half cross-validation with 20 random iterations to assess if biotypes in each half showed similar deviations for the optimal *k* clustering, incorporating stratification by diagnosis, sex, and site[23].

We performed permutation testing to evaluate the biotype stability through examining their null distribution that conducted out in TDC samples to eliminate disease-related variability[22]. The TDC individuals (n = 708) were randomly selected to a real TDC group (45% of 708) and a pseudo-ADHD group (55% of 708) with permutating 100 times followed by HYDRA analysis. These findings were then compared with clustering outcomes derived from the real-ADHD group: we assigned analogously-sized TDC and ADHD groups in permutation testing and repeated 100 times to ensure exacting the same sample size. The ARIs derived from the alternative scenarios were compared with these from null distribution using two-sample *t*-test to determine statistical significance of the *k* subtypes (p<.05 for a 2-tailed test).

We additionally calculated cluster-wise margin distances and soft assignment entropies. For each participant, we computed expression scores (SVM decision function values) from all k hyperplanes, then derived margin distances as the absolute expression scores normalized by the corresponding weight vector norms (|score|/||w||), representing the standardized distance from each hyperplane. Soft assignment probabilities were obtained by applying softmax transformation to the expression scores, converting raw SVM outputs into probabilistic cluster memberships. Assignment entropy was calculated as the Shannon entropy of these soft probabilities ($H = -\Sigma p_i \times log_2(p_i)$), quantifying the uncertainty in cluster assignment for each individual. Finally, we computed cluster-specific statistics by aggregating margin distances and assignment entropies for participants assigned to each subtype using the original HYDRA hard assignments.

Regarding the linear mixed-effect models that we employed to examine longitudinal changes in ADHD symptoms across biotypes, these models incorporated time, biotype, and their interaction as fixed effects, with age and sex as covariates. To account for within-subject correlations in repeated measurements, we specified random intercepts for each participant. We fitted

© 2026 Pan N et al. *JAMA Psychiatry*.

the model using the Newton-Raphson method (maximum iterations: 1,000; convergence tolerance: 1×10^{-8}) and set statistical significance at $p<.05$.

Specially, the development of mood disorders (e.g., anxiety and depression) among ADHD participants was evaluated through Kiddie Schedule for Affective Disorders and Schizophrenia interviews.

7. Contextualization Analysis

Group-averaged receptor maps from individual studies were registered to a standard Montreal Neurological Institute standard space template and parcellated based on the AAL atlas. In cases where multiple tracers targeted the same receptor, we used a weighted averaging procedure and normalized each receptor map across brain regions to obtain relative density distributions[24].

Meta-analytic task activation maps were derived from Neurosynth Compose (https://compose.neurosynth.org/) using coordinate-based multi-kernel density analysis (Chi-square workflow with association test maps) with FDR correction[25]. We selected activation maps corresponding to 123 cognitive terms, identified through their intersection with the Cognitive Atlas framework (as described in Hansen et al.)[26,27], and these terms encompassed a comprehensive range of neurocognitive processes. The maps were subsequently parcellated and normalized to construct a region-by-cognitive function matrix for each term. Neurosynth-derived probabilistic activation maps provided quantitative representations of region-specific neural activity patterns associated with distinct psychological processes.

Following Partial least squares (PLS) regression analysis with spin tests correcting for spatial autocorrelation (5,000 iterations), we then assessed the stability of cognitive term weights on the significant PLS component with highest explained variance by bootstrapping (10,000 iterations). Relative contributions of each cognitive term were quantified by calculating the ratio of term weights to their bootstrap-estimated errors (spin-derived $p_{spin}<.05$ and FDR-corrected).

8. Applying the Trained HYDRA Model to Validation Sample

During the training phase, the algorithm identifies discriminative hyperplanes through an iterative process. For each clustering solution *k*, the data is partitioned into 10-fold cross-validation sets. Within each fold, the algorithm estimates a set of support vector machine (SVM) hyperplanes, where each hyperplane is positioned in the high-dimensional feature space by its weight vector and bias term [28].

To validate the HYDRA model and assess the reproducibility of identified subtypes across different datasets, we applied the pre-trained model to validation data from the HBN datasets. During the adaptation process, hyperparameters of reference model were used as informed priors for the new dataset, and we adjusted the mean and variance within the latent Gaussian space before wrapping the adjusted data back to its original space[15]. A general linear model correction is also applied to remove potential confounding effects within validation cohort, following the same procedure used in the training phase. For pattern extraction, the trained HYDRA model is applied to the validation data to obtain expression scores across all dimensions [23]. These scores are computed using the preserved hyperplane weights (w_i) and bias terms (b_i) from the training phase, where each sample's expression score is calculated as

$$E_i = w_i X + b_i$$

Where X represent the feature matrix, and i-th dimension of the polytope (each dimension corresponds to a distinct linear SVM hyperplane). Subjects are then assigned to clusters based on their maximum expression scores across dimensions.

$$D = \underset{i}{\operatorname{argmax}}(E_i)$$

Due to the complexity of real-scenario (i.e., data distribution differences might alter distance score rankings), we then employed pattern alignment with greedy matching approach to correct potential misalignments and to provide more reliable results and better interpretability. For each clustering solution, cluster patterns are computed as the centroid of member samples' features. Pattern similarity between validation and reference clusters is quantified through pairwise Pearson correlations, yielding a correlation matrix. Cluster mapping is determined through a greedy matching approach, where each validation cluster is mapped to the reference cluster with which it shows the highest correlation.

eDiscussion

1. Parallel and Distinct Neural Dysfunctions in ADHD

Both parallel and distinct neural dysfunctions were identified in ADHD, linking response inhibition deficits to anterior cingulate-insular alterations and attention deficits to superior frontal gyrus changes[29]. In the action-mode network[30], the

© 2026 Pan N et al. *JAMA Psychiatry.*

anterior cingulate cortex orchestrates flexible cognitive control transitions and action monitoring through circuits with the insula[31], while the pallidum modulates frontostriatal inhibitory inputs[32]. These disruptions may disrupt the fine-tuned balance of inhibitory control, contributing to executive dysfunction in ADHD[33]. Conversely, the superior frontal gyrus embedding in the default mode network, shows dysregulation in ADHD that may impair sustained attention through interference hypothesis[34].

2. Future Directions for Clinical Translation

Several steps are required to translate our subtyping approach into clinical practice. First, validation studies using harmonized clinical protocols across populations are warranted. Second, prospective longitudinal studies should assess biotype stability, predictive validity, and preferential comorbidity emergence patterns. Third, controlled clinical trials are warranted to evaluate whether biotype-guided treatment selection improves outcomes, ultimately integrating into clinical decision support systems. For example, trials could evaluate whether Biotype 1 benefits from early adjunctive emotion regulation therapy combined with neuromodulation therapies targeting frontostriatal connectivity[35]. Biotype 2 may show superior response to medications that normalize cingulo-striatal abnormalities in action-mode network or targeted cognitive-behavioral interventions that leverage intact prefrontal control systems[36,37]. Biotype 3, with superior frontal gyrus alterations, may respond preferentially to attention training programs with neurofeedback to enhance task-positive network engagement[38].

© 2026 Pan N et al. *JAMA Psychiatry.*

Reference:

1. Gaser C, Dahnke R, Thompson PM, Kurth F, Luders E. CAT: A computational anatomy toolbox for the analysis of structural MRI data. *Gigascience*. 2024;13:1-13. doi:10.1093/gigascience/giae049
2. Ashburner J. A fast diffeomorphic image registration algorithm. *Neuroimage*. 2007;38(1):95-113. doi:10.1016/j.neuroimage.2007.07.007
3. Segal A, Parkes L, Aquino K, et al. Regional, circuit, and network heterogeneity of brain abnormalities in psychiatric disorders. *Nat Neurosci*. 2023;10(September):2022.03.07.22271986. doi:10.1038/s41593-023-01404-6
4. Tzourio-Mazoyer N, Landeau B, Papathanassiou D, et al. Automated anatomical labeling of activations in SPM using a macroscopic anatomical parcellation of the MNI MRI single-subject brain. *Neuroimage*. 2002;15(1):273-289. doi:10.1006/nimg.2001.0978
5. Wang H, Jin X, Zhang Y, Wang J. Single-subject morphological brain networks: Connectivity mapping, topological characterization and test-retest reliability. *Brain Behav*. 2016;6(4):1-21. doi:10.1002/brb3.448
6. Wang J, Wang X, Xia M, Liao X, Evans A, He Y. GRETNA: A graph theoretical network analysis toolbox for imaging connectomics. *Front Hum Neurosci*. 2015;9(JUNE):1-16. doi:10.3389/fnhum.2015.00386
7. Zhang J, Wang J, Wu Q, et al. Disrupted brain connectivity networks in drug-naive, first-episode major depressive disorder. *Biol Psychiatry*. 2011;70(4):334-342. doi:10.1016/j.biopsych.2011.05.018
8. Pan N, Qin K, Patino LR, et al. Aberrant brain network topology in youth with a familial risk for bipolar disorder: a task-based fMRI connectome study. *J Child Psychol Psychiatry*. 2024;65(8):1072-1086. doi:10.1111/jcpp.13946
9. Rubinov M, Sporns O. Complex network measures of brain connectivity: Uses and interpretations. *Neuroimage*. 2010;52(3):1059-1069. doi:10.1016/j.neuroimage.2009.10.003
10. Chen ZJ, He Y, Rosa-Neto P, Germann J, Evans AC. Revealing modular architecture of human brain structural networks by using cortical thickness from MRI. *Cereb Cortex*. 2008;18(10):2374-2381. doi:10.1093/cercor/bhn003
11. Fraza CJ, Dinga R, Beckmann CF, Marquand AF. Warped Bayesian linear regression for normative modelling of big data. *Neuroimage*. 2021;245(May):118715. doi:10.1016/j.neuroimage.2021.118715
12. Ge R, Yu Y, Qi YX, et al. Normative modelling of brain morphometry across the lifespan with CentileBrain: algorithm benchmarking and model optimisation. *Lancet Digit Heal*. 2024;6(3):e211-e221. doi:10.1016/S2589-7500(23)00250-9
13. Bayer JMM, Dinga R, Kia SM, et al. Accommodating site variation in neuroimaging data using normative and hierarchical Bayesian models. *Neuroimage*. 2022;264(May):119699. doi:10.1016/j.neuroimage.2022.119699
14. Parkes L, Moore TM, Calkins ME, et al. Transdiagnostic dimensions of psychopathology explain individuals' unique deviations from normative neurodevelopment in brain structure. *Transl Psychiatry*. 2021;11(1). doi:10.1038/s41398-021-01342-6

© 2026 Pan N et al. *JAMA Psychiatry.*

15. Rutherford S, Barkema P, Tso IF, et al. Evidence for embracing normative modeling. *Elife*. 2023;12. doi:10.7554/eLife.85082
16. Fraza C, Sønderby IE, Boen R, Shi Y, Beckmann CF, Marquand AF. Unraveling the link between CNVs, cognition and individual neuroimaging deviation scores from a population-based reference cohort. *Nat Ment Heal*. Published online 2024:1-13.
17. Marquand AF, Kia SM, Zabihi M, Wolfers T, Buitelaar JK, Beckmann CF. Conceptualizing mental disorders as deviations from normative functioning. *Mol Psychiatry*. 2019;24(10):1415-1424. doi:10.1038/s41380-019-0441-1
18. Burt JB, Helmer M, Shinn M, Anticevic A, Murray JD. Generative modeling of brain maps with spatial autocorrelation. *Neuroimage*. 2020;220(February):117038. doi:10.1016/j.neuroimage.2020.117038
19. He H, Sui J, Du Y, et al. Co-altered functional networks and brain structure in unmedicated patients with bipolar and major depressive disorders. *Brain Struct Funct*. 2017;222(9):4051-4064. doi:10.1007/s00429-017-1451-x
20. Li YO, Adali T, Calhoun VD. Estimating the number of independent components for functional magnetic resonance imaging data. *Hum Brain Mapp*. 2007;28(11):1251-1266. doi:10.1002/hbm.20359
21. Liu S, Wang H, Song M, et al. Linked 4-Way Multimodal Brain Differences in Schizophrenia in a Large Chinese Han Population. *Schizophr Bull*. 2019;45(2):436-449. doi:10.1093/schbul/sby045
22. Chand GB, Dwyer DB, Erus G, et al. Two distinct neuroanatomical subtypes of schizophrenia revealed using machine learning. *Brain*. 2020;143(3):1027-1038. doi:10.1093/brain/awaa025
23. Wen J, Fu CHY, Tosun D, et al. Characterizing Heterogeneity in Neuroimaging, Cognition, Clinical Symptoms, and Genetics among Patients with Late-Life Depression. *JAMA Psychiatry*. 2022;79(5):464-474. doi:10.1001/jamapsychiatry.2022.0020
24. Hansen JY, Shafiei G, Markello RD, et al. Mapping neurotransmitter systems to the structural and functional organization of the human neocortex. *Nat Neurosci*. 2022;25(11):1569-1581. doi:10.1038/s41593-022-01186-3
25. Kent J, Lee N, Peraza J, et al. Neurosynth Compose: A Free an Open Platform for Precise Large-Scale Neuroimaging Meta-Analysis. *Biol Psychiatry*. 2024;95(10):S156-S157. doi:10.1016/j.biopsych.2024.02.376
26. Poldrack RA, Kittur A, Kalar D, et al. The cognitive atlas: toward a knowledge foundation for cognitive neuroscience. *Front Neuroinform*. 2011;5:17. doi:10.3389/fninf.2011.00017
27. Luppi AI, Singleton SP, Hansen JY, et al. Contributions of network structure, chemoarchitecture and diagnostic categories to transitions between cognitive topographies. *Nat Biomed Eng*. Published online 2024. doi:10.1038/s41551-024-01242-2
28. Varol E, Sotiras A, Davatzikos C. HYDRA: Revealing heterogeneity of imaging and genetic patterns through a multiple max-margin discriminative analysis framework. *Neuroimage*. 2017;145:346-364.

© 2026 Pan N et al. *JAMA Psychiatry*.

doi:10.1016/j.neuroimage.2016.02.041
29. Hwang S, Meffert H, Parsley I, et al. Segregating sustained attention from response inhibition in ADHD: An fMRI study. *NeuroImage Clin*. 2019;21:101677. doi:10.1016/j.nicl.2019.101677
30. Dosenbach NUF, Raichle ME, Gordon EM. The brain's action-mode network. *Nat Rev Neurosci*. 2025;26(3):158-168. doi:10.1038/s41583-024-00895-x
31. Long Y, Pan N, Yu Y, et al. Shared and Distinct Neurobiological Bases of Bipolar Disorder and Attention-Deficit/Hyperactivity Disorder in Children and Adolescents: A Comparative Meta-Analysis of Structural Abnormalities. *J Am Acad Child Adolesc Psychiatry*. 2024;63(6):586-604. doi:10.1016/j.jaac.2023.09.551
32. Tomasi D, Volkow ND. Abnormal functional connectivity in children with attention-deficit/hyperactivity disorder. *Biol Psychiatry*. 2012;71(5):443-450. doi:10.1016/j.biopsych.2011.11.003
33. Hoogman M, Muetzel R, Guimaraes JP, et al. Brain imaging of the cortex in ADHD: A coordinated analysis of large-scale clinical and population-based samples. *Am J Psychiatry*. 2019;176(7):531-542. doi:10.1176/appi.ajp.2019.18091033
34. Koirala S, Grimsrud G, Mooney MA, et al. Neurobiology of attention-deficit hyperactivity disorder: historical challenges and emerging frontiers. *Nat Rev Neurosci*. 2024;25(December):759-775. doi:10.1038/s41583-024-00869-z
35. Lenzi F, Cortese S, Harris J, Masi G. Neuroscience and Biobehavioral Reviews Pharmacotherapy of emotional dysregulation in adults with ADHD : A systematic review and meta-analysis. *Neurosci Biobehav Rev*. 2018;84(June 2017):359-367. doi:10.1016/j.neubiorev.2017.08.010
36. Kowalczyk OS, Mehta MA, O'Daly OG, Criaud M. Task-Based Functional Connectivity in Attention-Deficit/Hyperactivity Disorder: A Systematic Review. *Biol psychiatry Glob open Sci*. 2022;2(4):350-367. doi:10.1016/j.bpsgos.2021.10.006
37. Dang C, Zhu Y, Luo X, et al. The promoting effects of digital targeted cognitive training in medication treatment for children with ADHD: a randomized controlled trial. *BMC Med*. 2025;23(1):371. doi:10.1186/s12916-025-04192-x
38. Rubia K, Criaud M, Wulff M, et al. Functional connectivity changes associated with fMRI neurofeedback of right inferior frontal cortex in adolescents with ADHD. *Neuroimage*. 2019;188:43-58. doi:10.1016/j.neuroimage.2018.11.055

© 2026 Pan N et al. *JAMA Psychiatry.*

Supplementary Tables and Figures

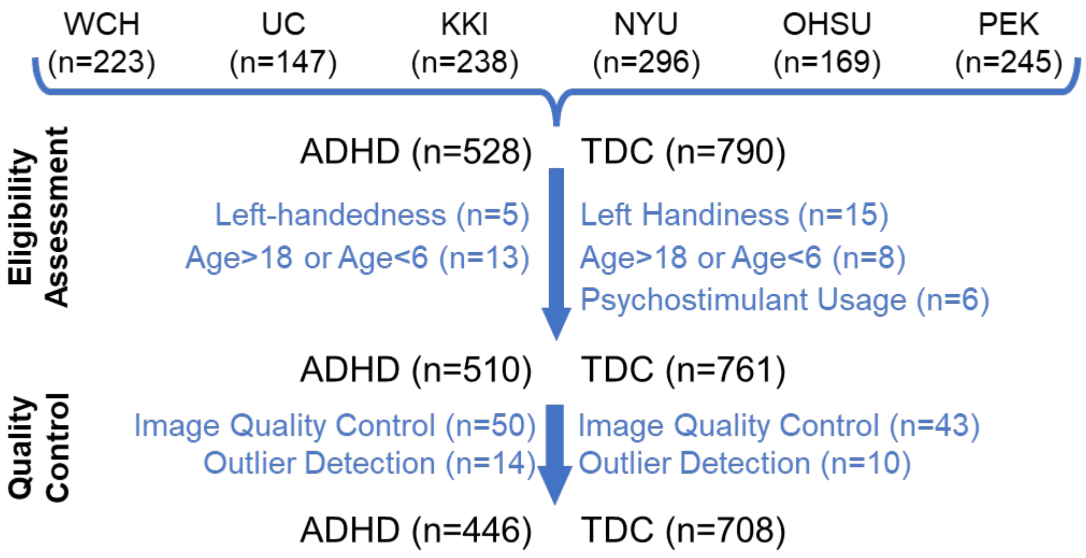

eFigure 1. Sample Selection Flowchart for Discovery Cohort.

Abbreviation: KKI = Kennedy Krieger Institute, NYU = New York University Langone Medical Center, OHSU = Oregon Health & Science University, PEK = Institute of Mental Health of Peking University, UC = University of Cincinnati, WCH = West China Hospital of Sichuan University.

Note: Subjects who appeared in multiple datasets (particularly KKI, NYU, and OHSU) across both ADHD-200 and ABIDE were included only once in the ADHD-200 analysis in the beginning to avoid data redundancy. Subjects with missing basic demographic information or imaging data were excluded prior to selection procedure. All subjects meet the criteria that IQ>70.

© 2026 Pan N et al. *JAMA Psychiatry.*

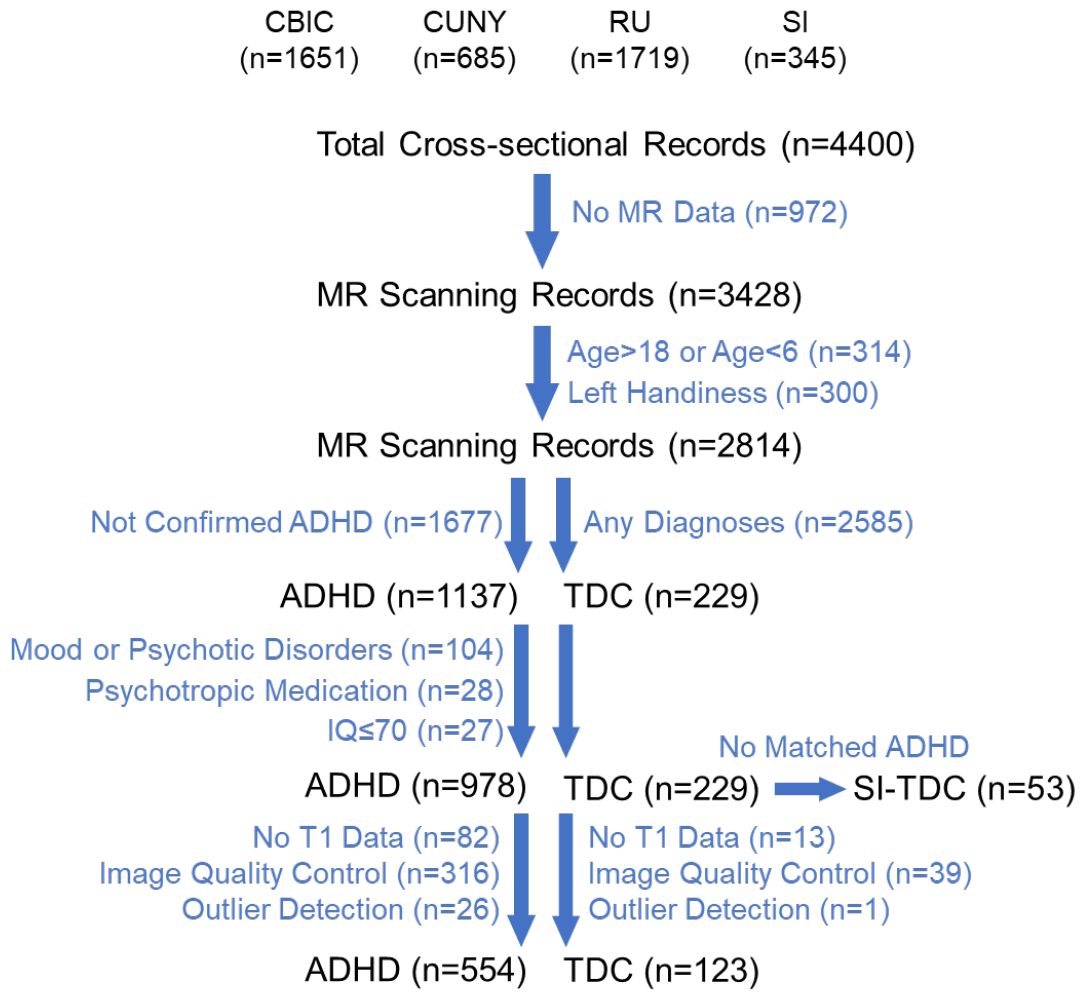

eFigure 2. Sample Selection Flowchart for Validation Cohort.

Abbreviation: RU = Rutgers University; CBIC = Cornell Brain Imaging Center; CUNY = City University of New York; SI = Staten Island Flagship Research Center.

Note: We included only ADHD participants with "confirmed" ADHD diagnoses recorded in Diagnosis_ClinicianConsensus instrument (Release 11), indicating that full DSM criteria were met and HBN's evaluation protocols were sufficient for diagnosis. The TDC samples in the validation cohort only served as adaptation data to account for site-specific variations and to fine-tune our pre-trained normative models using a transfer learning approach. TDC samples from the SI site were excluded from the validation cohort, as this site had no ADHD subjects for validation. For the ADHD group in HBN, only participants with confirmed diagnoses meeting full criteria were included, while excluding those with IQ≤70, history of mood or psychotic disorders, or prior psychotropic medication use.

© 2026 Pan N et al. *JAMA Psychiatry.*

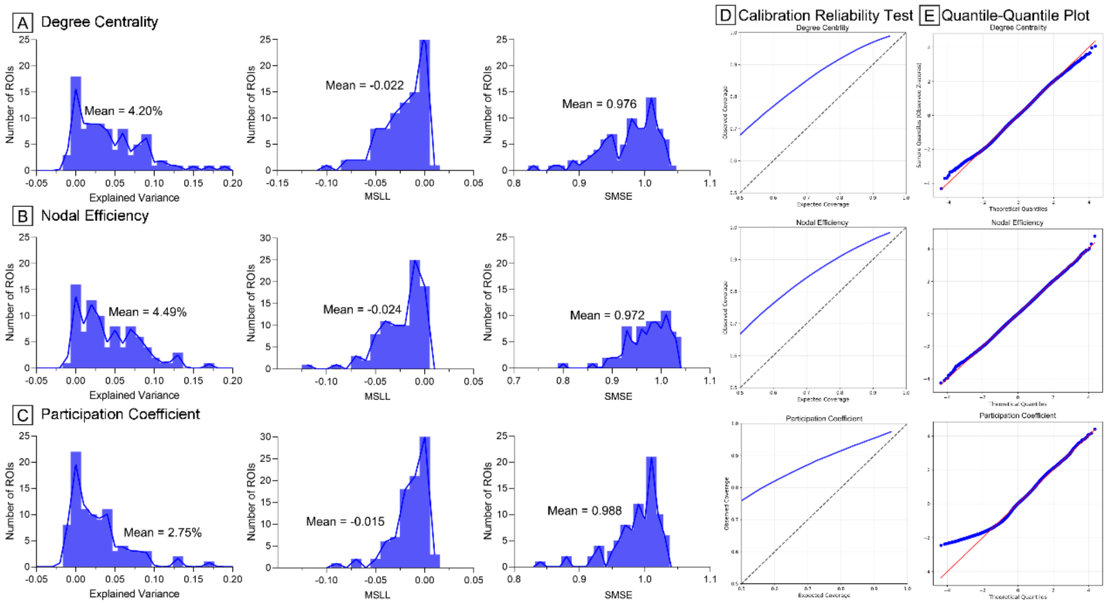

eFigure 3. Performance Metrics across Nodes for Normative Models.

Note: We present performance metrics of explained variance, mean standardized log loss, and standardized mean squared error for the training data. D) Deviations from the diagonal line indicating departures from normality that could suggest model misspecification or the presence of systematic biases in predictions. E) Points falling on the diagonal line indicate perfect calibration, while systematic deviations suggest that the model's uncertainty estimates are over- or underconfident.

© 2026 Pan N et al. *JAMA Psychiatry.*

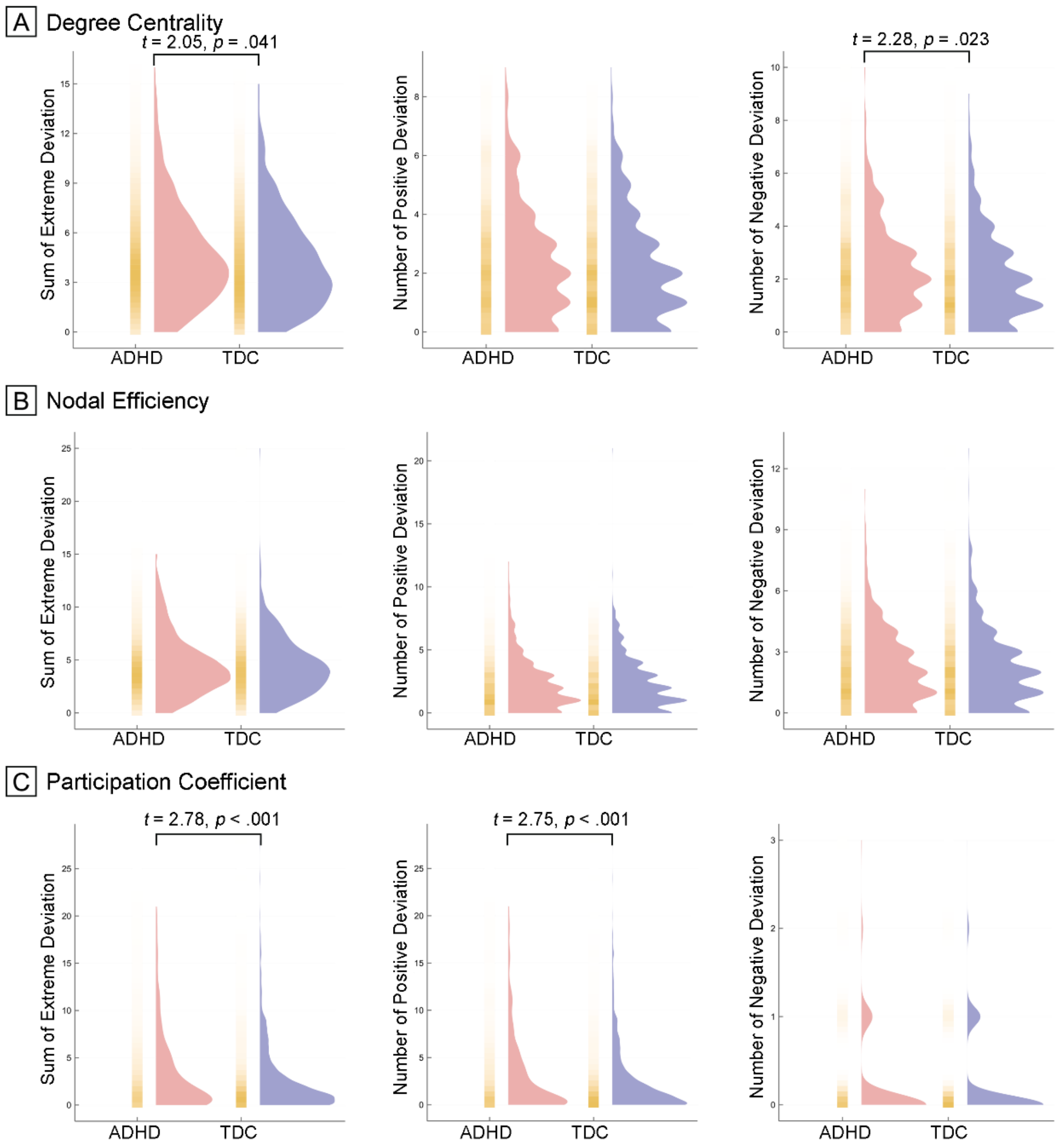

eFigure 4. Number of Extreme Deviations across Groups.

Note: To summarize the nodal heterogeneity of extreme deviations in each group, the deviations of topological patterns were thresholded at Z≥|2.0| for visualization, and we computed the proportion of children with suprathreshold positive or negative deviations (i.e., atypically increased or reduced phenotypes) in each brain node.

© 2026 Pan N et al. *JAMA Psychiatry.*

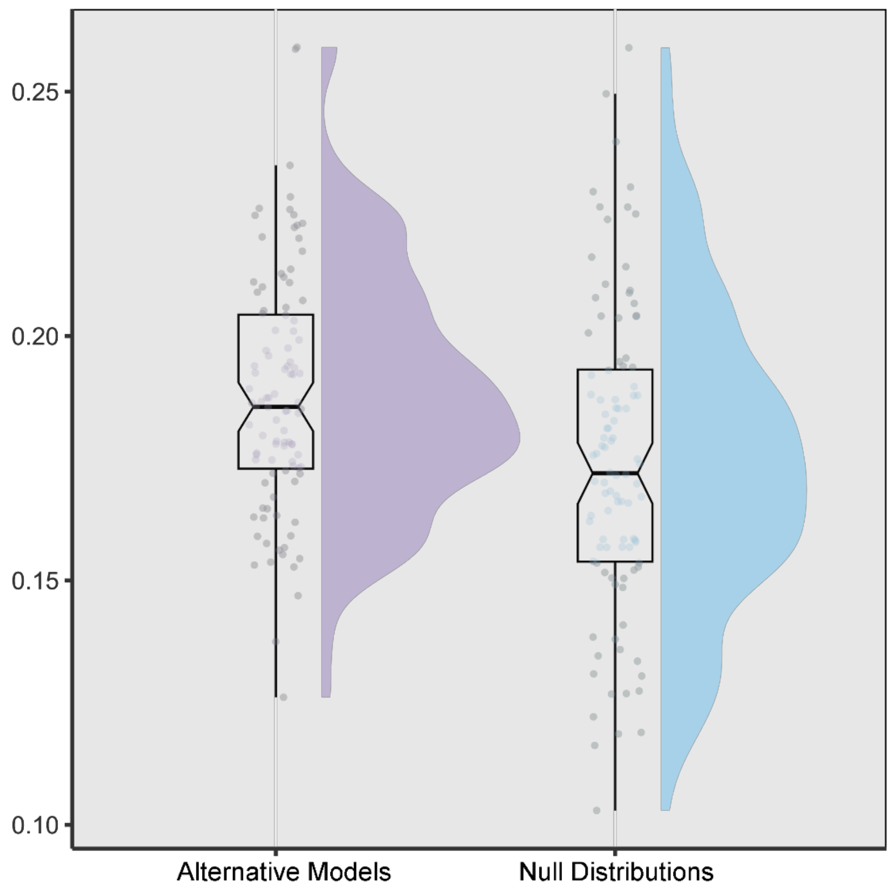

eFigure 5. Permutation Testing for Statistical Significance of Clustering.

Note: null distributions were derived from TDC and pseudo-ADHD samples, and alternative models were generated from analogously-sized TDC and ADHD groups with repeating 100 times for each distribution. $ARI_{alternative}$ vs. ARI_{null} = 0.188 vs. 0.174, t=3.42, p<.001.

© 2026 Pan N et al. *JAMA Psychiatry.*

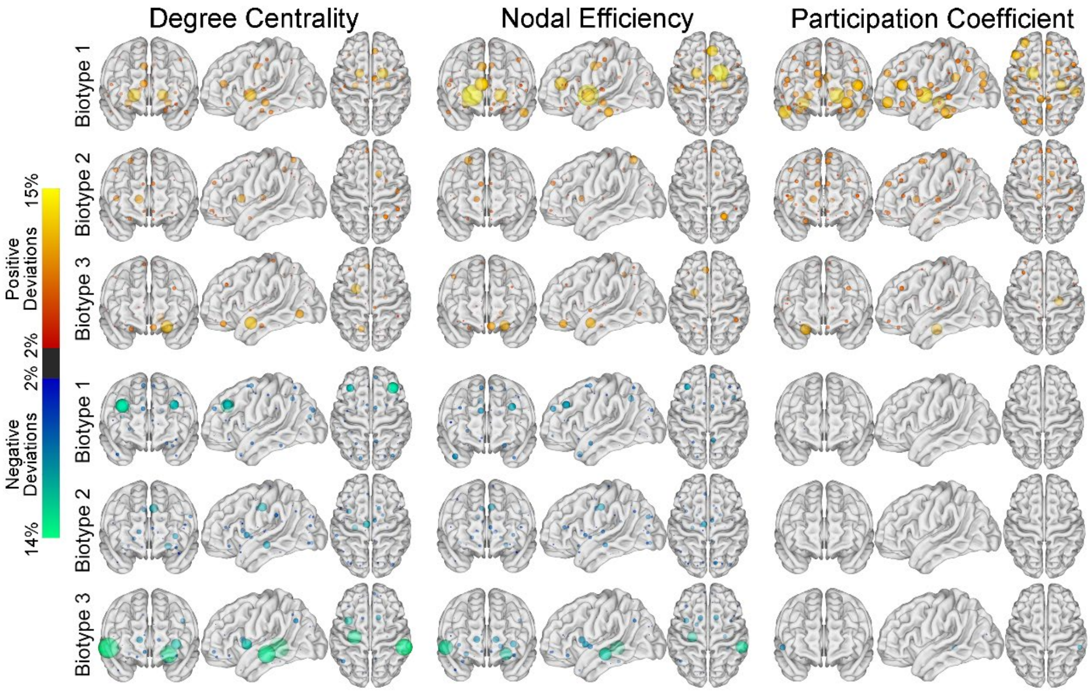

eFigure 6. Extreme Deviation Patterns among Biotypes in Boys.

Note: The ARI of this HYDRA model for *k*=3 is 0.160. Biotype 1, 2, and 3 consisted of 106, 132, and 101 ADHD boys. The red-yellow patterns indicate atypically increased topological phenotypes in relative to normative models, and the blue-green patterns indicate atypically reduced topological phenotypes.

© 2026 Pan N et al. *JAMA Psychiatry*.

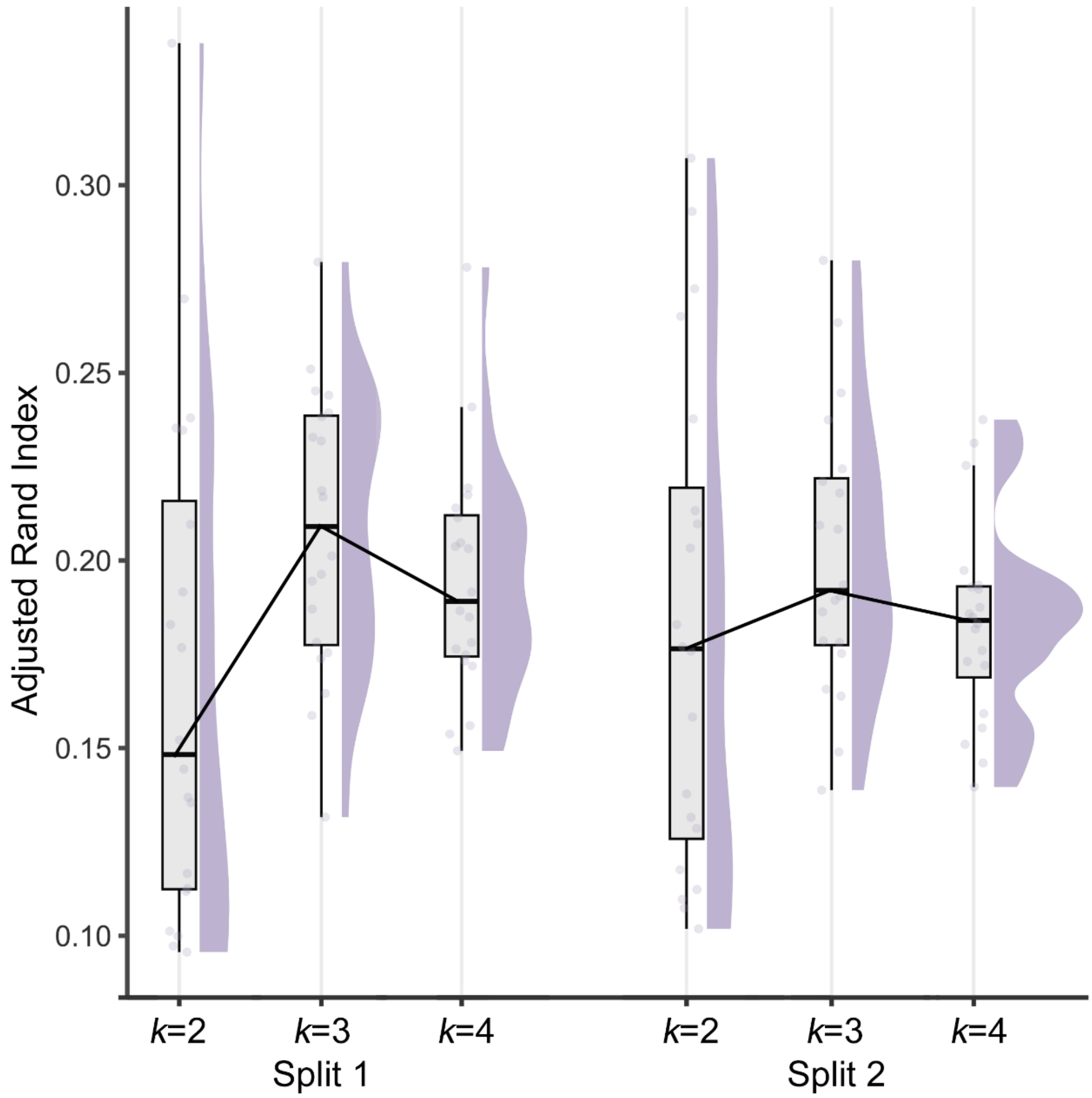

eFigure 7. Adjusted Rand Indices in Split-half Cross-validation.

Note: the split-half cross-validation was performed by incorporating stratification by sex and site with 20 random iterations. Higher ARI indicates higher reproducibility for the cluster solution. In our study, k=3 also yielded to the optimal solution in split-half cross-validation based on ARIs (Split 1: 0.208±0.038, Split 2: 0.201±0.037). In the contrast, Split 1: 0.169 and Split 2: 0.182 for k=2, while Split 1: 0.194 and Split 2: 0.183 for k=4.

© 2026 Pan N et al. *JAMA Psychiatry.*

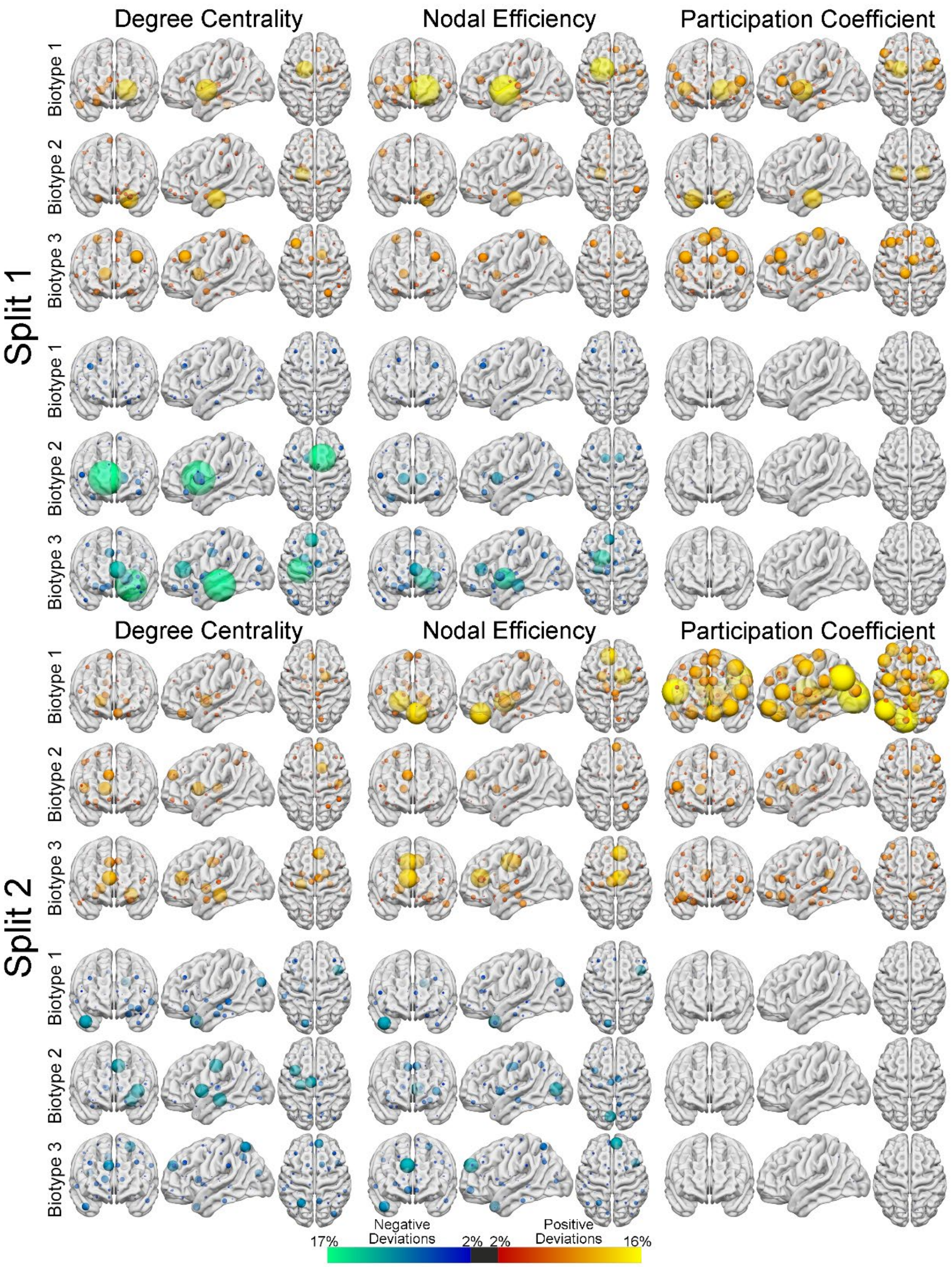

eFigure 8. Stratified Split-half Cross-validation for Clustering.

Note: Biotype 1, 2, and 3 consisted of 84, 73, and 69 ADHD children in split 1, and Biotype 1, 2, and 3 consisted of 69, 86, and 65 ADHD children in split 2. The red-yellow patterns indicate atypically increased topological phenotypes in relative to normative models, and the blue-green patterns indicate atypically reduced topological phenotypes.

© 2026 Pan N et al. *JAMA Psychiatry.*

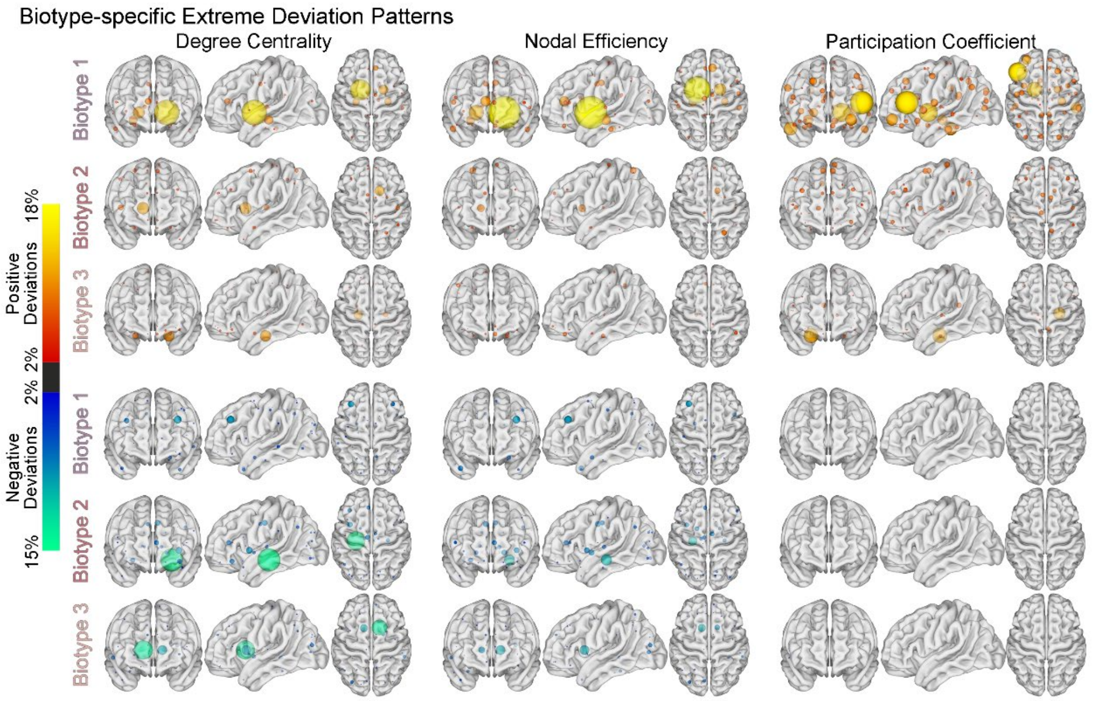

eFigure 9. Biotype-specific Extreme Deviation Patterns.

Note: Biotype-specific extreme deviation maps were generated by computing the proportion of subjects with suprathreshold deviations. Red-yellow nodes represent atypically increased topological metrics, while blue-green nodes indicate decreased metrics. Nodes exhibiting significant between-biotype differences across all modalities were highlighted with black squares, in accordance with our proposed segregation model.

© 2026 Pan N et al. *JAMA Psychiatry.*

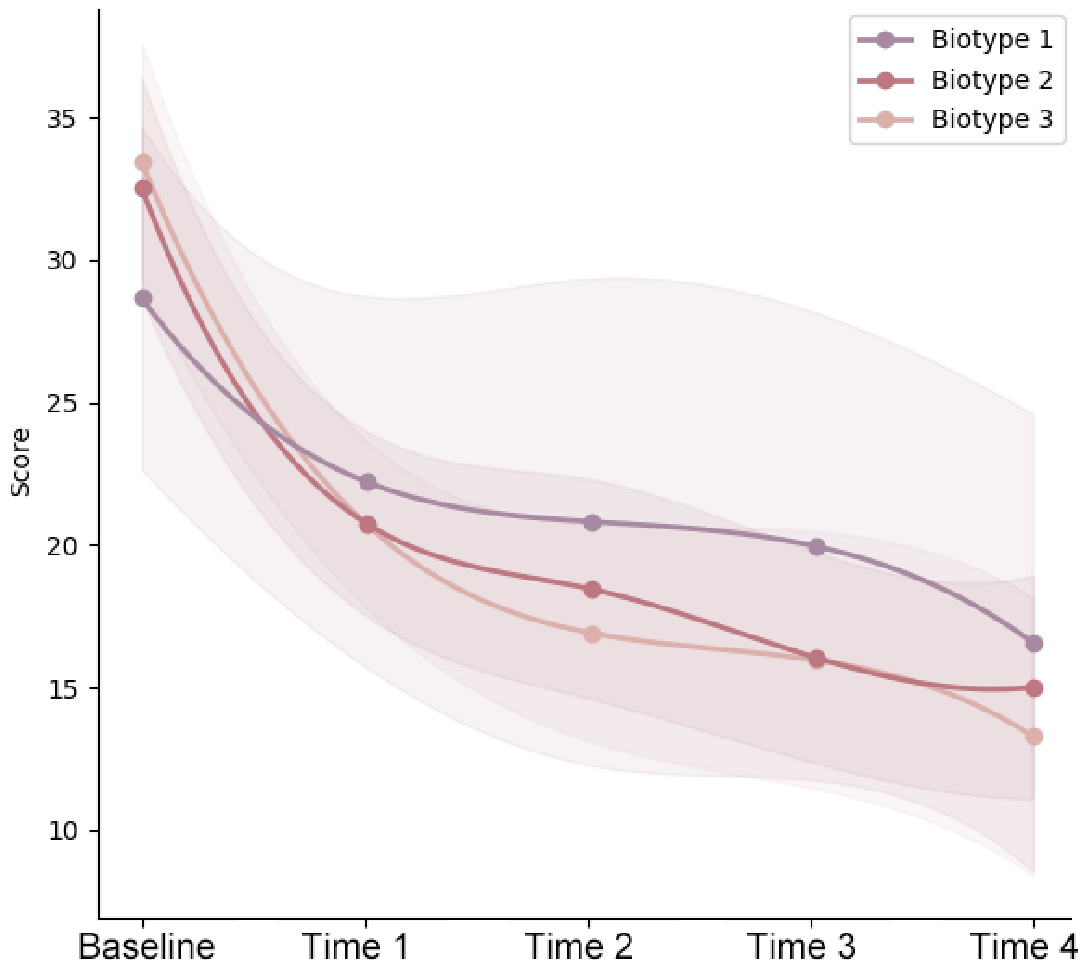

eFigure 10. Biotype-specific Longitudinal Trajectories of Deficient Emotional Self-regulation.

Note: We evaluated the changes of deficient emotional self-regulation profiles based on Child Behavioral Checklist (total scores of attention, aggression, and anxious/depressed subscales) annually over four years. Time-by-biotype interactions: Biotype 1 vs. 2: Z=-2.11, p=.035; Biotype 1 vs. 3: Z=-2.66, p=.008.

© 2026 Pan N et al. *JAMA Psychiatry.*

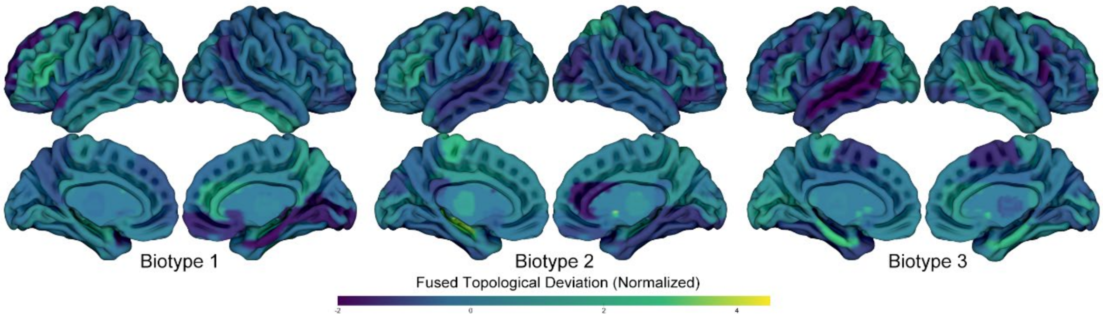

eFigure 11. Fused Topological Deviations across Biotypes.

Note: To comprehensively characterize brain network organization of ADHD, we developed an indicator termed as fused topological deviations by aggregating case-control difference maps in extreme deviations across three modalities, capturing network centrality, integration, and segregation for each brain node. The 19 neurotransmitter density maps were derived from 1,308 healthy participants in 31 different PET studies.

© 2026 Pan N et al. *JAMA Psychiatry*.

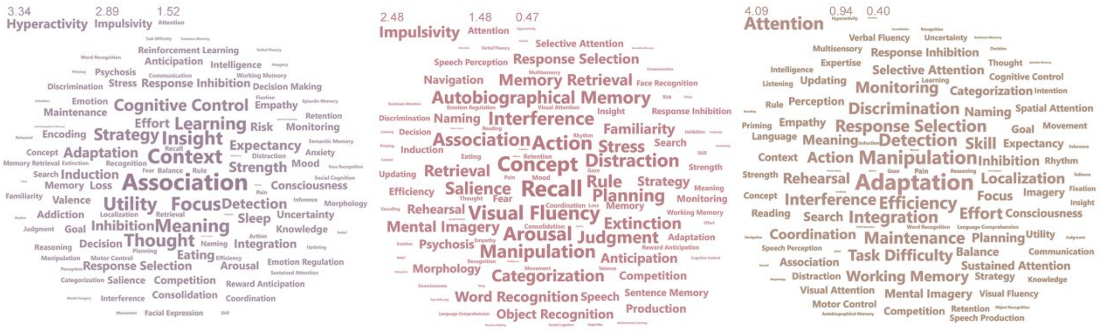

eFigure 12. Biotype-specific Functional Correlates.

Note: Cognitive terms derived from Neurosynth meta-analyses, scaled by their respective Z-scores of PLS1 loading across biotypes. Word size is proportional to absolute Z-score magnitude. Core ADHD clinical dimensions (attention, hyperactivity, and impulsivity) are highlighted at the top.

© 2026 Pan N et al. *JAMA Psychiatry.*

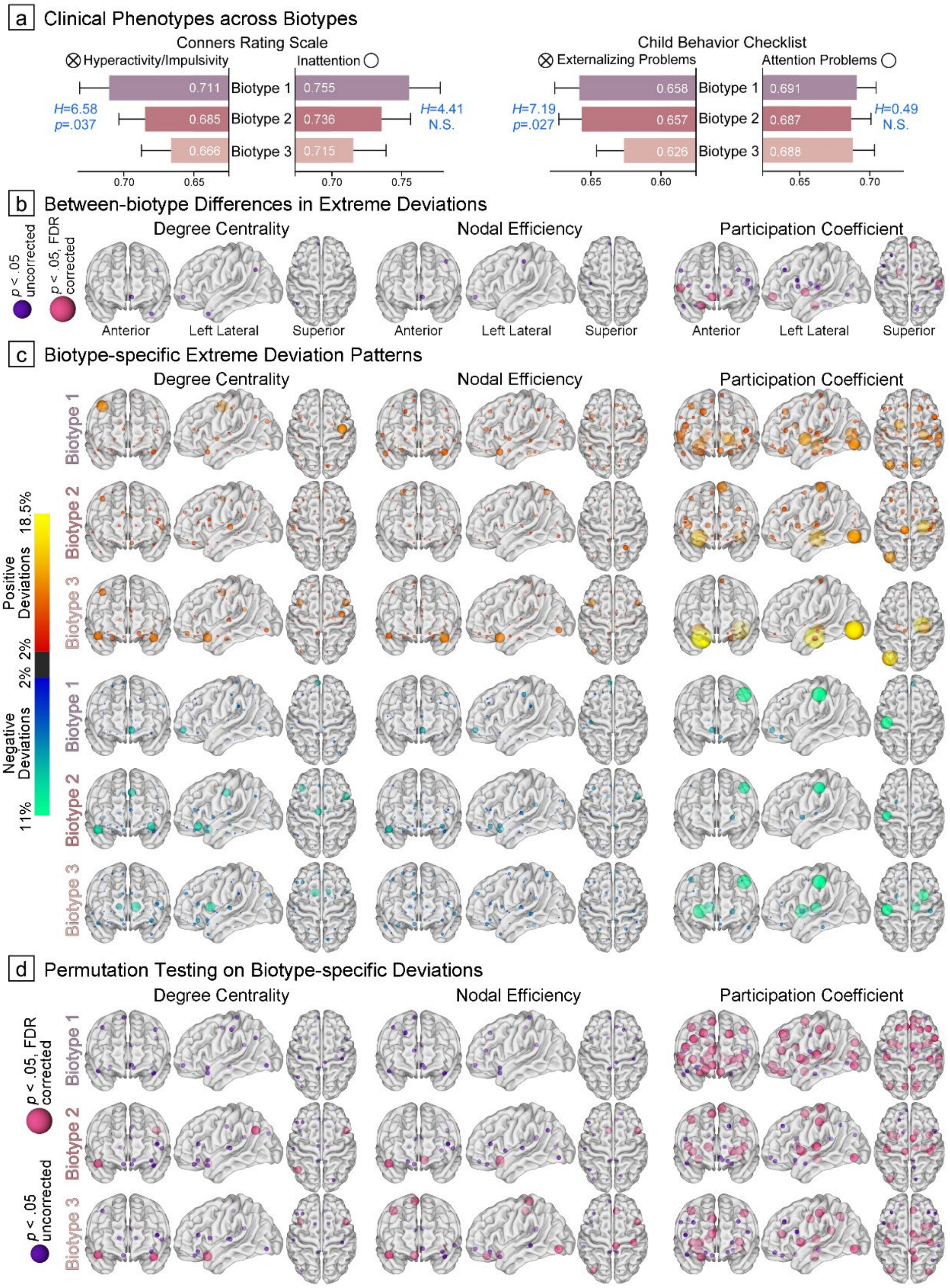

eFigure 13. Biotype Validation Based on Pre-trained HYDRA Model.

(a) and (b), Between-biotype differences in symptom severity (checkmarks indicated replicated findings) and atypical neural mechanisms. Locations of hub regions were exhibited, with smaller purple nodes indicating significance at $p_{uncorreced}$<.05 and larger pink nodes indicating significance at p_{FDR}<.05. (c), Biotype-specific extreme deviation maps were generated by computing the proportion of subjects with suprathreshold deviations. Red-yellow nodes represent atypically increased topological metrics, while blue-green nodes indicate the opposite. (d), Hub nodes that exhibited statistical significance when compared to null patterns.

© 2026 Pan N et al. *JAMA Psychiatry.*

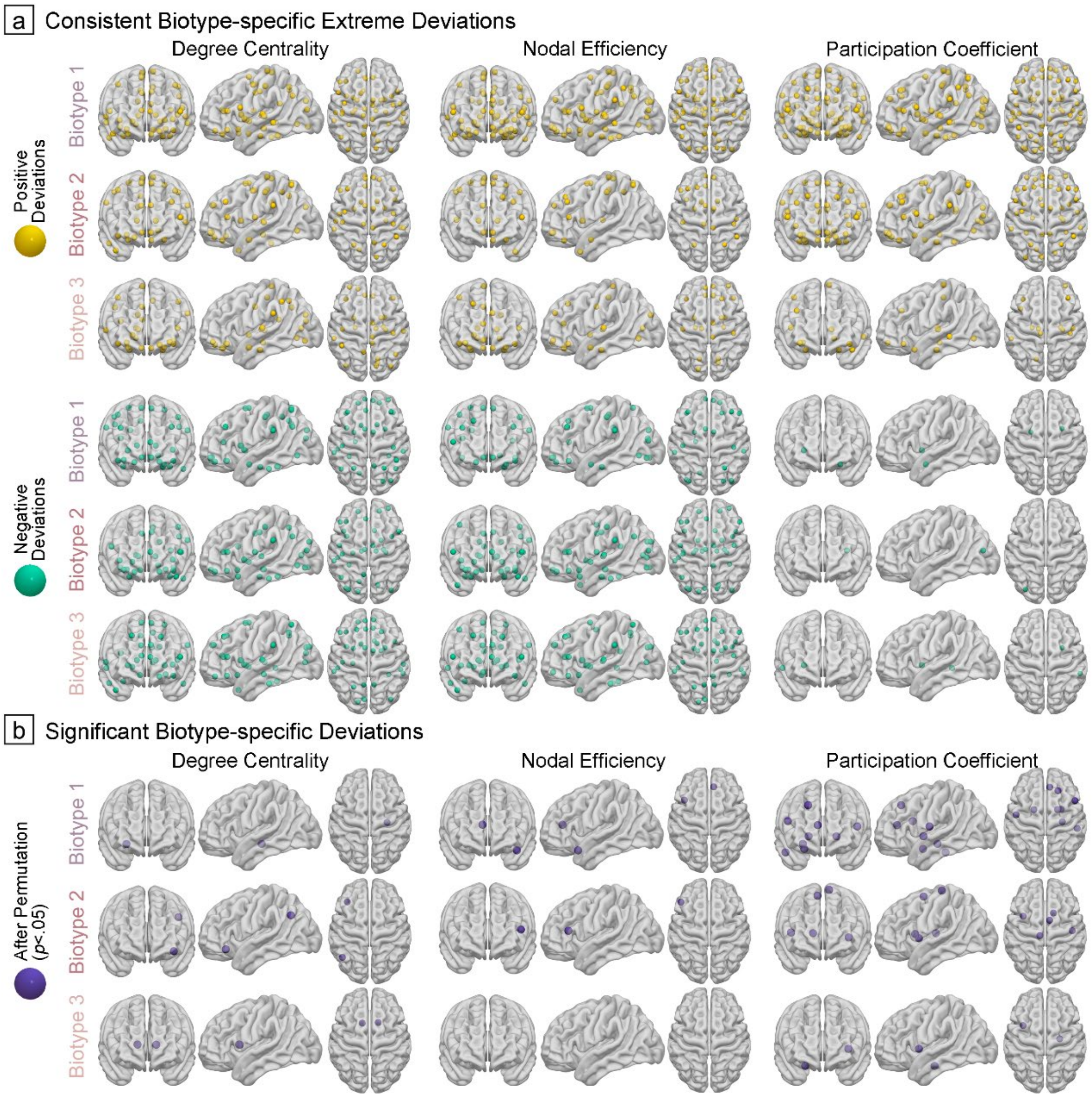

eFigure 14. Cross-Cohort Consistency of Hub Alterations.

Note: (a) Biotype-specific extreme deviation maps display nodes showing consistent alterations across both discovery and validation cohorts. Orange nodes represent regions with atypically increased topological metrics, while indigo nodes indicate decreased metrics. (b) Hub regions demonstrating statistical significance (p<.05) in group-based permutation tests compared to null patterns. Both panels exclusively present nodes exhibiting extreme deviations in both discovery and validation cohorts, highlighting the robust and replicable neurobiological markers of ADHD biotypes.

© 2026 Pan N et al. *JAMA Psychiatry.*

eTable 1. Inclusion and Exclusion Criteria across Sites in Discovery Cohort.

Sites	Evaluation (Diagnosis & ADHD measure & IQ)	ADHD - Inclusion	ADHD - Exclusion	TDC - Inclusion	TDC - Exclusion	Preparation of MRI scanning
**WCH**	Structured Clinical Interview for DSM-IV (SCID) Patient and Non-Patient Edition; Conners' Parent Symptom Questionnaire and Child Behavior Checklist; Wechsler Intelligence Scale for Chinese Children-Revised (WISCC-R).	1. Diagnosis of ADHD;<br>2. Aged from 6-18 years old;<br>3. Full scale IQ of 90 or higher;<br>4. Right-handedness.	1. History of any major Axis I psychiatric comorbid disorder, head trauma, neurologic disorder, or neurosurgery;<br>2. Current or past treatment with psychotropic medication;<br>3. Any systemic illness that might affect brain anatomy and function;<br>4. Contraindications to MR scan.		1. Any major Axis I psychiatric diagnoses;<br>2. History of receiving psychotropic medication or having first-degree relatives with a known history of psychiatric illness.	
**UC**	Schedule of Affective Disorders and Schizophrenia for Children—Present and Lifetime Version (KSADS-PL); ADHD Rating Scale (ADHD-RS) IV; Wechsler Abbreviated Scale of Intelligence (WASI).	1. Diagnosis of ADHD;<br>2. Aged from 10-18 years old;<br>3. Full scale IQ of 80 or higher;<br>4. Right-handedness.	1. Exposure to psychostimulants or other ADHD medications for at least 3 months prior to screening; lifetime exposure to mood stabilizers or antipsychotic medications; exposure to psychotropic medication during the 30 days prior to screening;<br>2. Contraindications to MR scan;<br>3. History of major medical or neurological illness or a significant episode of loss of consciousness;<br>4. Lifetime DSM-5 substance use disorder and currently comorbid mood, conduct, eating or psychotic disorders.			
**KKI-ADHD-200**	Diagnostic Interview for Children and Adolescents, Fourth Edition (DICA-IV, 1997); Conners' Parent Rating Scale-Revised, Long version (CPRS-LV); DuPaul ADHD Rating Scale-IV(Reid, 1998); Wechsler Intelligence Scale for Children-Fourth Edition (WISC-IV).	1. Diagnosis of ADHD: the DICA-IV and either had a T-score of 65 or greater on the CPRS-R Long Form (DSM-IV Inattentive) and/or M (DSM-IV Hyperactive/Impulsive) or met criteria on the DuPaul ADHD Rating Scale IV<br>2. Full Scale IQ of 80 or higher.	1. History of language disorder/reading disability, hearing impairment, or history of other neurological or psychiatric disorder (DSM-IV diagnoses other than Oppositional Defiant Disorder or Specific Phobias).<br>2. History of taking psychoactive medications other than stimulants.	T-scores of 60 or below on the DSM-IV Inattention (L) and DSM-IV Hyperactivity (M) subscales of CPRS-LV.	1. History of behavioral, emotional, or serious medical problems;<br>2. History of school-based intervention services;<br>3. Current diagnosis of DSM-IV psychiatric disorder except specific phobia as reported on the DICA-IV.	Children who were taking stimulant medication were removed from these medications the day before and the day of testing.

© 2026 Pan N et al. *JAMA Psychiatry*.

**KKI-ABIDE I&II**		-		1. Aged 8-12 years old;<br>2. Informed consent provided by a parent or guardian and assent provided by the child;<br>3. Full Scale IQ of 80 or higher.	1. History of a definitive neurologic disorder including seizures (except for uncomplicated brief febrile seizures), tumor, severe head injury, stroke, lesion; Major visual impairment; Contraindications to MR scan<br>2. Psychiatric conditions (ABIDE I): Diagnoses other than Simple Phobia; Had any immediate family members with autism spectrum disorders<br>3. Medical history: Trauma at birth (ABIDE I only); Severe chronic medical disorder (ABIDE II only)<br>4. Additional exclusion criteria (ABIDE II only): History of alcohol/substance abuse or dependency; Current psychotropic medication or meeting criteria for diagnosis of a learning disability; Reached developmental level of 3 or above on the Physical Development Scale	
**NYU-ADHD-200**	KSADS-PL; CPRS-LV; WASI.	1.Diagnosis of ADHD based on parent and child responses to the KSADS-PL as well as on a T-score greater than or equal to 65 on at least one ADHD related index of the CPRS-R: LV;<br>2. Full scale IQ of 80 or higher;<br>3. Right-handedness.	History of other chronic medical conditions.	1. Absence of any Axis-I psychiatric diagnoses per parent and child KSADS-PL interview;<br>2. T-scores below 60 for all the CPRS-R: LV ADHD summary scales.	History of any Axis-I psychiatric diagnoses, and other chronic medical conditions.	Psychostimulant drugs were withheld at least 24 hours before scanning.
**NYU-ABIDE I&II**		-		Absence of any current Axis-I disorders based on the KSADS-PL and based on the SCID-I/NP and ACDS interviews for adults.	1. Current chronic systemic medical conditions;<br>2. Contraindications to MRI scanning, pregnancy;<br>3. Use of antipsychotics.	

© 2026 Pan N et al. *JAMA Psychiatry*.

**OHSU-ADHD-200**	Kiddie Schedule for Affective Disorders and Schizophrenia (KSADS-I) administered to a parent; parent and teacher Connors' Rating Scale-3rd Edition; WISC-IV&V.	Diagnosis of ADHD.	1. History of neurological illness, chronic medical problems, sensorimotor handicap, autistic disorder, mental retardation, or significant head trauma (with loss of consciousness).<br>2. Evidence of psychotic disorder or bipolar disorder.		Presence of ADHD, conduct disorder, major depressive disorder, or history of psychotic disorder.	Children prescribed short-acting stimulant medications were scanned after a minimum washout of five half-lives (i.e., 24-48 hours depending on the preparation).
**OHSU-ABIDE-I&II**		-			1. History of neurological illness/impairment; intellectual disability/mental retardation;<br>2. Evidence of psychotic disorder; autistic spectrum disorder; significant head trauma (with loss of consciousness); bipolar disorder; major depressive disorder (ABIDE-II specifies "currently experiencing a major depressive episode"); chronic medical problems, sensorimotor handicap and conduct disorder (ABIDE-I only); prescribed long-acting psychotropic medications, seizure, other major medical conditions and substance abuse (ABIDE-II only).<br>3. IQ<80, ABIDE-II specifies IQ<70	
**PKU-ADHD-200**	KSADS-PL; ADHD-RS-IV; WISCC-R.	1. Diagnosis of ADHD;<br>2. Right-handedness;<br>3. Full Scale IQ of 80 or higher.	Lifetime history of head trauma with loss of consciousness, neurological disease and diagnosis of either schizophrenia, affective disorder, pervasive development disorder, or substance abuse.			Psychostimulant medications were withheld at least 48 hours prior to scanning.

Note: Among these, KKI, NYU, OHSU, and PKU datasets were retained and derived from the ADHD-200 initiative after excluding sites that had fewer than ten subjects per group or did not use 3.0 Tesla scanners. For more details about ADHD-200 and ABIDE-I&II datasets, please see https://fcon_1000.projects.nitrc.org/indi/adhd200/ and https://fcon_1000.projects.nitrc.org/indi/abide/. Since the HBN initiative launched by the Child Mind Institute is considered as transdiagnostic datasets in pediatric mental health (no specific inclusion and exclusion criteria were provided), we set our criteria as we demonstrated in Figure S3 to ensure the consistency with our discovery cohort. For more information about HBN, please see https://fcon_1000.projects.nitrc.org/indi/cmi_healthy_brain_network/index.html. To reconcile the heterogeneity across sites, we further excluded children with: 1) left-handedness; 2) age>18 or age<6; 3) psychostimulant usage in TDC. Subjects who appeared in multiple datasets (particularly KKI, NYU, and OHSU) across both ADHD-200 and ABIDE were included only once in the ADHD-200 analysis to avoid data redundancy.

© 2026 Pan N et al. *JAMA Psychiatry.*

eTable 2. Included PET Studies on Neurotransmitter Distribution.

Receptor	System	Tracer	N	Measure
5HT1a	Serotonin	[*carbonyl*-11C]WAY-100635	35	BPnd
5HT1b	Serotonin	[11C]P943	65	BPnd
5HT1b	Serotonin	[11C]P943	23	BPnd
5HT2a	Serotonin	[11C]CIMBI-36	29	Bmax
5HT4	Serotonin	[11C]SB207145	59	Bmax
5HT6	Serotonin	[11C]GSK215083	30	BPnd
5HTT	Serotonin	[11C]DASB	18	BPnd
5HTT	Serotonin	[11C]DASB	100	Bmax
a4b2	Acetylcholine	[18F]FLUBATINE	30	Vt
CB1	Cannabinoid	[11C]OMAR	77	Vt
D1	Dopamine	[11C]SCH23390	13	BPnd
D2	Dopamine	[11C]FLB457	37	BPnd
D2	Dopamine	[11C]FLB457	55	BPnd
DAT	Dopamine	[123I]FP-CIT	174	SUVR
GABAa	GABA	[11C]FLUMAZENIL	6	Vt
GABAabz	GABA	[11C]FLUMAZENIL	16	Bmax
H3	Histamine	[11C]GSK189254	8	Vt
M1	Acetylcholine	[11C]LSN3172176	24	BPnd
NMDAR	Glutamate	[18F]GE-179	10	Vt
NMDAR	Glutamate	[18F]GE-179	10	Vt
NMDAR	Glutamate	[18F]GE-179	9	Vt
mGluR5	Glutamate	[11C]ABP688	22	BPnd
mGluR5	Glutamate	[11C]ABP688	28	BPnd
mGluR5	Glutamate	[11C]ABP688	73	BPnd
MOR	Opioid	[11C]CARFENTANIL	39	BPnd
MOR	Opioid	[11C]CARFENTANIL	204	BPnd
NET	Norepinephrine	[11C]MRB	10	BPnd
NET	Norepinephrine	[11C]MRB	77	BPnd
VAChT	Acetylcholine	[18F]FEOBV	4	SUVR
VAChT	Acetylcholine	[18F]FEOBV	5	SUVR
VAChT	Acetylcholine	[18F]FEOBV	18	SUVR

Note: This table is derived from Table 1 in the paper of Hansen et al [24].

© 2026 Pan N et al. *JAMA Psychiatry.*

eTable 3. Demographic and Phenotypical Measures of Datasets across Sites.

Sites	Number	Age (years)	Sex (F/M)	Ethnicity/Race	TIV (cm^3)	IQ [a]	Subtype (C/I/H) [b]	Inattention [c]	Hyperactivity/ Impulsivity [c]
WCH	ADHD (92)	10.53±2.37	14/78	Asian:92	1505.26±132.85	105.33±14.53	53/37/2	10.96±2.96	6.19±3.06
	TDC (100)	10.95±2.43	19/81	Asian:100	1501.99±121.60	114.17±12.92	-	3.61±3.24	2.07±2.01
UC	ADHD (90)	13.58±2.34	31/59	Black/African:26<br>Latino/Hispanic:2<br>Mixed:7<br>White/Caucasian:55	1439.62±135.16	106.06±12.40	52/37/1	20.60±5.20	14.50±8.10
	TDC (45)	14.24±2.19	16/29	Black/African:9<br>Asian:4<br>Mixed:3<br>White/Caucasian:29	1453.17±136.52	115.38±12.43	-	3.33±4.03	1.98±2.33
KKI	ADHD (20)	10.28±1.62	10/10	Unspecified:20	1477.24±129.30	106.15±15.16	15/4/1	74.35±10.63	74.15±10.14
	TDC (197)	10.36±1.23	77/120	Unspecified:197	1550.85±146.82	113.68±10.77	-	45.54±5.05	46.85±4.66
NYU	ADHD (114)	11.32±2.81	31/83	Unspecified:114	1450.61±134.65	105.59±14.15	72/41/1	71.37±9.15	68.42±12.35
	TDC (113)	11.66±3.09	45/68	Unspecified:113	1453.94±145.66	113.79±13.53	-	46.03±6.31	46.45±5.26
OHSU	ADHD (38)	9.06±1.19	10/28	Unspecified:38	1471.13±120.75	108.95±13.63	22/13/3	72.50±8.23	69.84±13.43
	TDC (116)	9.79±1.59	64/52	Unspecified:116	1468.09±133.48	116.78±12.75	-	47.38±6.33	46.32±6.92
PKU	ADHD (92)	12.12±2.06	11/81	Asian:92	1494.53±117.32	105.24±13.05	32/59/1	27.72±4.19	21.97±6.49
	TDC (137)	11.38±1.85	58/79	Asian:137	1472.31±125.57	117.79±13.30	-	15.84±3.99	13.61±3.69
RU	ADHD (165)	10.21±2.81	50/115	Unspecified:165	1456.49±146.33	98.25±14.75	66/88/11	58.59±10.01	57.62±10.99
	TDC (66)	10.04±2.68	30/36	Unspecified:66	1475.19±140.27	100.68±16.92	-	54.60±11.45	55.17±10.84
CUNY	ADHD (80)	9.84±2.96	28/52	Unspecified:80	1507.79±156.12	106.73±14.02	42/32/6	56.10±10.88	56.90±10.83
	TDC (12)	9.95±2.57	3/9	Unspecified:12	1489.23±138.97	98.90±16.35	-	55.95±11.15	56.41±10.58
CBIC	ADHD (309)	10.09±2.72	104/205	Unspecified:309	1502.51±139.30	100.95±15.77	134/158/17	56.75±11.27	56.66±10.74
	TDC (45)	9.95±2.61	20/25	Unspecified:45	1496.63±145.68	101.31±16.82	-	54.62±11.54	55.01±11.04

Abbreviation: F/M, female/male; TIV, total intracranial volume; IQ, intelligence quotient; WCH, West China Hospital of Sichuan University; UC, University of Cincinnati; KKI, Kennedy Krieger Institute; NYU, New York University; OHSU, Oregon Health & Science University; PEK, Peking university. Notes: Data are presented as mean ± standard

© 2026 Pan N et al. *JAMA Psychiatry.*

deviation unless otherwise indicated. [a] IQ measure: WCH&PKU - Wechsler Intelligence Scale for Chinese Children-Revised (WISCC-R); UC&NYU - Wechsler Abbreviated Scale of Intelligence (WASI); KKI - Wechsler Intelligence Scale for Children, Fourth and Fifth Edition (WISC-IV&V); OHSU – WASI and WISC-IV. [b] ADHD Subtype: C = ADHD – combined type; I = ADHD – inattentive; H = ADHD - hyperactive/impulsive. [c] ADHD Measure: KKI&NYU - Conners' Parent Rating Scale-Revised, Long version; OHSU - Connors' Rating Scale-3rd Edition; WCH - Connors' Rating Scale-2nd Edition, Child Behavior Checklist; UC&PKU - ADHD Rating Scale IV.

© 2026 Pan N et al. *JAMA Psychiatry*.

eTable 4. Scan Parameters of Datasets across Sites.

sites	Field Strength (Tesla)	Channel	TR/TE (ms/ms)	Flip angle	Matrix	FOV	Slice Thickness (mm)	Slice Number
WCH	3	12	1900/2.5	9	256×256	256×256	1	176
	3	12	8.5/3.4	12	256×256	256×256	1	156
UC	3	NA	8.1/3.7	8	256×224	256×224	1	160
KKI	3	8	8.0/3.7	8	256×200	256×200	1	200
	3	32	8.2/3.7	8	212×172	212×172	1	150
NYU	3	NA	2530/3.25	7	256×192	256×256	1.3	128
OHSU	3	12	2300/3.58	10	256×256	256×239	1.1	160
PKU	3	NA	2530/3.39	7	256×192	256×256	1.33	128
	3		2530/3.45	7	256×256	256×209	1	176
	3		2000/3.67	12	256×256	240×240	1	192
	3		1950/2.6	10	240×256	240×256	1.3	128
	3		2530/3.37	7	256×256	256×256	1.33	128
	3		1770/3.92	12	512×512	256×256	1	176
	3		845/2.89	8	256×256	261×261	1.3	144
RU	3	32	2500/3.15	8	320×320	256×256	0.8	224
CBIC	3	32	2500/3.15	8	320×320	256×256	0.8	224
CUNY	3	32	2500/3.15	8	320×320	256×256	0.8	224

Abbreviation: TR/TE, repetition time/echo time; FOV, field of view; WCH, West China Hospital of Sichuan University; UC, University of Cincinnati; KKI, Kennedy Krieger Institute; NYU, New York University; OHSU, Oregon Health & Science University; PKU, Peking university; RU, Rutgers University; CBIC, Cornell Brain Imaging Center; CUNY, City University of New York. Note: T1 weighted-Vnav data in validation cohort were excluded due to its difference of scanning parameters with traditional T1 weighted sequence.

© 2026 Pan N et al. *JAMA Psychiatry.*

eTable 5. Cross-site Validation of Observed Topological Deviations

Site	DC		NE		PC	
	ADHD	TDC	ADHD	TDC	ADHD	TDC
KKI	50.56	48.37	48.94	43.75	49.65	46.20
NYU	59.97	49.85	60.11	46.84	58.40	45.43
OHSU	54.02	46.15	51.08	47.57	51.59	48.22
PEK	57.70	45.88	51.76	48.25	53.42	46.87
UC	62.28	46.98	62.64	46.30	64.35	49.53
WCH	52.76	48.24	58.49	47.74	55.08	47.01

Note: The data presented here, mean balanced accuracy (%), near and below chance-level performance (50%) indicated the absence of site-related artifacts in the observed deviations.

© 2026 Pan N et al. *JAMA Psychiatry.*

eTable 6. Nodal Metrics with Significant Case-control Differences.

No. AAL	Label	Region	Extreme Positive Deviations (%)	Extreme Negative Deviations (%)	Observed Difference (%)	Cohen's *h*	Group-based Permutation		Spatial Permutation	
							p-value	p-value (FDR)	p-value	p-value (FDR)
Degree Centrality (|Z|≥2.0)										
72	**R. Caudate**	Subcortical	5.61	6.28	7.79	0.30	<.001	<.001	<.001	<.001
37	**L. Hippocampus**	Limbic lobe	4.04	6.95	6.33	0.24	<.001	<.001	<.001	<.001
39	L. ParaHippocampal	Limbic lobe	4.71	3.59	4.34	0.18	.002	.054	.000	.009
76	R. Pallidum	Subcortical	5.61	3.36	4.31	0.17	.004	.086	.000	.006
69	L. Paracentral lobule	Frontal lobe	3.36	2.47	3.00	0.15	.012	.209	.016	.180
75	L. Pallidum	Subcortical	6.05	2.69	3.66	0.15	.014	.213	.001	.025
15	L. Frontal_Inf_Orb	Frontal lobe	3.36	4.71	3.41	0.14	.017	.224	.004	.051
38	R. Hippocampus	Limbic lobe	4.71	3.36	3.41	0.14	.022	.246	.004	.051
61	L. Parietal_Inf	Parietal lobe	1.79	0.67	-2.76	-0.15	.025	.246	.024	.212
65	L. Angular	Parietal lobe	2.02	4.26	2.61	0.12	.040	.364	.041	.334
41	L. Amygdala	Limbic lobe	4.93	2.47	2.74	0.12	.070	.472	.018	.180
90	R. Temporal_Inf	Temporal lobe	4.26	2.02	2.46	0.11	.060	.472	.047	.353
Nodal Efficiency (|Z|≥2.0)										
37	L. Hippocampus	Limbic lobe	4.26	4.71	4.31	0.17	.004	.114	<.001	<.001
76	R. Pallidum	Subcortical	6.05	3.14	3.97	0.15	.009	.180	<.001	<.001
75	L. Pallidum	Subcortical	6.73	3.59	4.24	0.16	.010	.180	<.001	<.001
69	L. Paracentral lobule	Frontal lobe	4.26	2.69	4.13	0.20	.001	.090	.001	.018
72	R. Caudate	Subcortical	4.48	3.81	3.35	0.14	.027	.266	.002	.039
83	L. Temporal_Pole_Sup	Limbic lobe	0.67	2.02	-3.24	-0.16	.014	.198	.003	.039
61	L. Parietal_Inf	Parietal lobe	1.35	0.67	-3.49	-0.19	.003	.114	.004	.043
12	R. Frontal_Inf_Oper	Frontal lobe	2.02	0.90	-3.16	-0.16	.015	.198	.004	.043
13	L. Frontal_Inf_Tri	Frontal lobe	3.36	3.36	2.91	0.13	.037	.335	.008	.082
3	L. Frontal_Sup	Frontal lobe	1.12	1.35	-2.76	-0.15	.024	.266	.014	.124
10	R. Frontal_Mid_Orb	Frontal lobe	1.35	1.57	-2.45	-0.12	.053	.395	.027	.204

© 2026 Pan N et al. *JAMA Psychiatry.*

38	R. Hippocampus	Limbic lobe	3.81	3.14	2.43	0.11	.083	.450	.027	.204
64	R. SupraMarginal	Parietal lobe	0.90	1.57	-2.48	-0.13	.046	.373	.030	.209
33	L. Cingulum_Mid	Limbic lobe	2.91	3.14	2.38	0.11	.063	.404	.034	.219
48	R. Lingual	Occipital lobe	2.02	1.35	-2.43	-0.12	.062	.404	.042	.240
Participant Coefficient (|Z|≥2.0)										
13	**L. Frontal_Inf_Tri**	Frontal lobe	7.17	1.57	5.50	0.24	<.001	<.001	<.001	<.001
9	**L. Frontal_Mid_Orb**	Frontal lobe	6.73	0.90	4.52	0.20	.001	.045	<.001	<.001
29	L. Insula	Central region	4.71	0.00	-3.77	-0.15	.016	.391	<.001	.012
80	R. Heschl	Temporal lobe	4.26	0.00	2.42	0.14	.018	.391	.023	.234
19	L. Supp_Motor_Area	Frontal lobe	4.93	0.00	2.39	0.13	.030	.391	.036	.328
51	L. Occipital_Mid	Occipital lobe	3.81	2.02	2.72	0.13	.034	.391	.022	.234
1	L. Precentral	Central region	4.48	0.00	2.22	0.12	.036	.391	.047	.353
50	R. Occipital_Sup	Occipital lobe	3.59	0.45	2.06	0.12	.043	.391	.085	.431
77	L. Thalamus	Subcortical	5.61	0.00	2.50	0.12	.045	.391	.020	.234
8	R. Frontal_Mid	Frontal lobe	5.16	0.45	2.50	0.12	.047	.391	.023	.234
75	L. Pallidum	Subcortical	5.38	0.00	2.42	0.12	.048	.391	.017	.234
69	L. Paracentral lobule	Frontal lobe	5.61	0.00	2.22	0.11	.069	.480	.046	.353
68	R. Precuneus	Parietal lobe	5.83	1.79	2.68	0.11	.075	.483	.020	.234
Degree Centrality (|Z|≥2.6)										
37	L. Hippocampus	Limbic lobe	0.67	1.56	1.96	0.19	.002	.198	.094	.999
7	L. Frontal_Mid	Frontal lobe	0.00	0.00	2.13	0.18	.004	.198	.076	.999
12	R. Frontal_Inf_Oper	Frontal lobe	1.35	1.35	-1.55	-0.25	.010	.288	.183	.999
Nodal Efficiency (|Z|≥2.6)										
38	R. Hippocampus	Limbic lobe	2.24	0.00	1.67	0.15	.015	.948	.114	.988
72	R. Caudate	Subcortical	1.35	0.45	1.70	0.13	.031	.948	.103	.988
47	L. Lingual	Occipital lobe	1.12	1.57	1.37	0.14	.032	.948	.278	.988
Participant Coefficient (|Z|≥2.6)										
9	L. Frontal_Mid_Orb	Frontal lobe	2.69	0.00	1.98	0.16	.010	.864	.042	.999
36	R. Cingulum_Post	Limbic lobe	0.90	0.00	0.90	0.19	.023	.999	.383	.999

© 2026 Pan N et al. *JAMA Psychiatry.*

Note: We identified brain regions with significant case-control differences in nodal metrics in either group-based or spatial permutation tests (*p*-value uncorrected < .050), and brain regions survived FDR correction (*q*-value < .050) in both group-based permutation tests and spatial permutation tests were shown in bold type when thresholding at $|Z| \geq 2.0$. The observed differences were obtained by subtracting the extreme deviation maps of TDC from those of children with ADHD. These maps represent the proportion of individuals showing an extreme deviation in a given brain region, thresholded at $|Z| \geq 2.0$ and 2.6, respectively.

© 2026 Pan N et al. *JAMA Psychiatry*.

eTable 7. Brain Patterns of Joint Component of Topological Metrics

Metrics	Label	No. AAL	R/L	Region	Independent Source (*S*)	*p*-values
Degree Centrality	Frontal_Mid	8	Right	Frontal lobe	2.27	.023
	ParaHippocampal	40	Right	Limbic lobe	-2.62	.009
	ParaHippocampal	39	Left	Limbic lobe	-2.56	.011
	Pallidum	76	Right	Subcortical	-2.29	.022
	Rectus	27	Left	Frontal lobe	-2.11	.035
Nodal Efficiency	Rectus	27	Left	Frontal lobe	-3.15	.002
	Lingual	47	Left	Occipital lobe	-2.36	.018
	Pallidum	76	Right	Subcortical	-2.03	.042
Participant Coefficient	Frontal_Inf_Oper	12	Right	Frontal lobe	2.39	.017
	Rectus	28	Right	Frontal lobe	2.31	.021
	Cuneus	45	Left	Occipital lobe	2.19	.029
	Occipital_Inf	53	Left	Occipital lobe	2.03	.042
	Putamen	73	Left	Subcortical	-2.16	.031

Note: Brain regions were identified at |Z| > 2.0 of normalized independent source (*S*) for visualization. Brain regions significantly observed in at least two modalities were in bold type.

© 2026 Pan N et al. *JAMA Psychiatry.*

eTable 8. Cluster-Wise Margin Distance and Assignment Entropy.

	Assignment Entropy	Margin Distance			Soft Membership Probability		
		Margin 1	Margin 2	Margin 3	Biotype 1	Biotype 2	Biotype 3
Biotype 1	1.47±0.09	1.89±0.93	1.07±0.75	0.80±0.59	0.49±0.08	0.25±0.07	0.26±0.08
Biotype 2	1.49±0.08	1.00±0.70	1.56±0.82	0.81±0.65	0.26±0.07	0.47±0.08	0.27±0.08
Biotype 3	1.48±0.08	0.91±0.67	0.96±0.77	1.51±0.59	0.25±0.06	0.25±0.06	0.49±0.07

Note: Higher margin distance means greater distance from decision boundary, indicating more confident classification, and lower assignment entropy means more concentrated soft assignment probabilities, indicating more reliable classification

© 2026 Pan N et al. *JAMA Psychiatry*.

eTable 9. Between-biotype Differences across Topological Modalities.

No. AAL	Label of Brain Region	Extreme Deviations in Biotype 1 (%)	Extreme Deviations in Biotype 2 (%)	Extreme Deviations in Biotype 3 (%)	χ² statistic	*p*-value	*p*-value (FDR)
Degree Centrality							
**75**	**L. Pallidum**	16.2	7.34	2.36	16.80	<.001	.020
80	R. Heschl	1.41	7.91	0.79	13.51	.001	.043
**32**	**R. Cingulum_Ant**	9.15	0.56	6.30	13.11	.001	.043
37	L. Hippocampus	10.56	16.38	3.94	11.75	.003	.063
85	L. Temporal_Mid	7.75	3.39	0.00	11.12	.004	.069
8	R. Frontal_Mid	9.86	3.95	2.36	8.57	.014	.206
**4**	**R. Frontal_Sup**	1.41	2.26	7.09	7.75	.021	.258
40	R. ParaHippocampal	1.41	3.39	7.87	7.55	.023	.258
7	L. Frontal_Mid	9.86	7.34	2.36	6.18	.045	.454
Nodal Efficiency							
**32**	**R. Cingulum_Ant**	17.61	9.04	3.94	14.06	<.001	.080
**75**	**L. Pallidum**	11.27	2.26	5.51	11.33	.003	.156
12	R. Frontal_Inf_Oper	6.34	2.26	9.96	9.96	.007	.192
37	L. Hippocampus	11.27	11.86	9.53	9.53	.009	.192
**4**	**R. Frontal_Sup**	4.23	1.13	8.70	8.70	.013	.232
7	L. Frontal_Mid	9.86	6.21	8.10	8.10	.017	.261
72	R. Caudate	3.52	11.86	7.24	7.24	.027	.334
18	R. Rolandic_Oper	8.45	2.82	6.52	6.52	.038	.334
44	R. Calcarine	0.70	6.21	6.40	6.40	.041	.334
Participation Coefficient							
**75**	**L. Pallidum**	13.38	2.82	0.00	27.34	<.001	.001
90	R. Temporal_Inf	11.27	1.13	2.36	20.23	<.001	.002
76	R. Pallidum	9.15	1.69	0.79	16.39	<.001	.008
13	L. Frontal_Inf_Tri	15.49	6.78	3.94	12.64	.002	.041

© 2026 Pan N et al. *JAMA Psychiatry*.

**32**	**R. Cingulum_Ant**	6.34	0.00	2.36	12.16	.002	.041
40	R. ParaHippocampal	2.82	4.52	11.81	10.75	.005	.057
65	L. Angular	7.75	1.13	2.36	10.74	.005	.057
50	R. Occipital_Sup	8.45	2.26	1.57	10.57	.005	.057
64	R. SupraMarginal	8.45	3.39	0.79	10.20	.006	.061
89	L. Temporal_Inf	8.45	3.95	0.79	9.38	.009	.075
17	L. Rolandic_Oper	8.45	2.82	1.57	9.25	.010	.075
**4**	**R. Frontal_Sup**	9.15	2.82	2.36	9.22	.010	.075
15	L. Frontal_Inf_Orb	6.34	0.56	3.94	8.26	.016	.102
9	L. Frontal_Mid_Orb	12.68	6.21	3.94	8.10	.017	.102
56	R. Fusiform	7.04	2.82	0.79	8.08	.017	.102
21	L. Olfactory	7.75	3.95	0.79	8.03	.018	.102
38	R. Hippocampus	9.86	2.82	4.72	7.63	.022	.115
55	L. Fusiform	6.34	2.26	0.79	7.54	.023	.115
20	R. Supp_Motor_Area	4.93	7.34	0.79	7.11	.029	.133
72	R. Caudate	3.52	6.78	0.79	7.00	.030	.133
52	R. Occipital_Mid	5.63	1.13	1.57	6.95	.031	.133
16	R. Frontal_Inf_Orb	6.34	1.13	3.15	6.60	.037	.147
29	L. Insula	8.45	2.82	3.15	6.52	.038	.147
62	R. Parietal_Inf	2.11	5.65	0.79	6.47	.039	.147
78	R. Thalamus	4.93	4.52	0.00	6.22	.045	.161

Note: Biotype 1, 2, and 3 consisted of 142, 177, and 127 ADHD children, respectively. Extreme deviations were defined as deviations of topological patterns exceeding the threshold of Z=|2.0|. Nodes that are consistently significant in three modalities were highlighted.

© 2026 Pan N et al. *JAMA Psychiatry*.

eTable 10. Comparison of Number of Extreme Deviations across Biotypes.

Number of Brain Regions		Degree Centrality	Nodal Efficiency	Participation Coefficient
Biotype 1	Extreme Positive/Negative Deviations	59/55	68/45	81/4
	Significant Hubs	9	13	23
Biotype 2	Extreme Positive/Negative Deviations	47/53	31/49	60/2
	Significant Hubs	9	8	9
Biotype 3	Extreme Positive/Negative Deviations	43/44	39/44	40/6
	Significant Hubs	6	3	2

Note: Extreme positive/negative deviations were defined as deviations of topological patterns exceeding the threshold of Z=|2.0|. Hubs demonstrated statistical significance in group-based permutation tests ($p_{uncorreced}$<.05).

© 2026 Pan N et al. *JAMA Psychiatry*.

eTable 11. Biotype-specific Extreme Deviation Patterns.

No. AAL	Brain Region	Extreme Positive Deviations (%)	Extreme Negative Deviations (%)	Observed Difference	Cohen's *h*	*p*-value	*p*-value (FDR)
Degree Centrality—Biotype 1							
75	**L. Pallidum**	15.49	0.70	11.12	0.37	<.001	<.001
76	R. Pallidum	10.56	2.11	8.02	0.29	<.001	.018
37	L. Hippocampus	9.15	1.41	5.9	0.23	.011	.302
41	L. Amygdala	4.23	5.63	5.2	0.20	.015	.302
38	R. Hippocampus	9.15	0.70	5.2	0.20	.017	.302
32	R. Cingulum_Ant	7.75	1.41	4.77	0.19	.024	.363
7	L. Frontal_Mid	1.41	8.45	4.78	0.18	.035	.407
40	R. ParaHippocampal	0.70	0.70	-4.52	-0.25	.036	.407
44	R. Calcarine	0.70	0.70	-3.82	-0.22	.046	.462
Degree Centrality—Biotype 2							
72	**R. Caudate**	10.73	3.39	10.02	0.36	<.001	<.001
37	L. Hippocampus	1.69	14.69	11.72	0.40	<.001	<.001
80	R. Heschl	7.34	0.56	4.66	0.21	.006	.168
60	R. Parietal_Sup	6.21	2.82	4.94	0.20	.010	.234
15	L. Frontal_Inf_Orb	2.82	6.78	4.94	0.19	.015	.270
32	R. Cingulum_Ant	0.56	0.00	-3.82	-0.27	.021	.321
38	R. Hippocampus	2.26	6.78	4.38	0.18	.028	.365
65	L. Angular	1.69	5.65	3.67	0.16	.037	.378
69	L. Paracentralobule	4.52	1.69	3.39	0.17	.038	.378
Degree Centrality—Biotype 3							
72	R. Caudate	0.79	13.39	10.07	0.36	<.001	<.001
39	L. ParaHippocampal	10.24	1.57	7.86	0.30	.001	.054
85	L. Temporal_Mid	0.00	0.00	-5.23	-0.46	.008	.246
12	R. Frontal_Inf_Oper	0.00	0.79	-4.86	-0.30	.019	.428
76	R. Pallidum	3.15	6.30	4.79	0.19	.032	.507
71	L. Caudate	0.00	9.45	4.79	0.19	.034	.507
Nodal Efficiency—Biotype 1							
75	**L. Pallidum**	17.61	0.00	11.54	0.37	<.001	<.001
32	R. Cingulum_Ant	9.86	1.41	6.89	0.26	.003	.108
37	L. Hippocampus	9.86	1.41	6.61	0.25	.004	.108
76	R. Pallidum	11.97	0.00	6.74	0.25	.005	.108
41	L. Amygdala	4.93	6.34	6.33	0.24	.006	.108
33	L. Cingulum_Mid	7.75	0.70	4.78	0.20	.015	.218
3	L. Frontal_Sup	0.70	0.00	-4.53	-0.29	.019	.218
69	L. Paracentral_Lobule	4.93	2.11	4.22	0.20	.019	.218
13	L. Frontal_Inf_Tri	5.63	2.82	4.64	0.20	.024	.238
44	R. Calcarine	0.70	0.00	-3.96	-0.27	.032	.270
83	L. Temporal_Pole_Sup	0.70	0.70	-4.52	-0.25	.033	.270
47	L. Lingual	2.82	4.93	4.21	0.19	.039	.293
38	R. Hippocampus	9.15	0.00	4.63	0.19	.042	.294
Nodal Efficiency—Biotype 2							
37	L. Hippocampus	1.69	10.17	7.2	0.27	<.001	.036
72	**R. Caudate**	8.47	3.39	6.92	0.25	.001	.054
69	L. Paracentral_Lobule	5.65	2.82	5.65	0.25	.002	.060

© 2026 Pan N et al. *JAMA Psychiatry.*

73	L. Putamen	0.56	0.00	-3.82	-0.27	.021	.428
60	R. Parietal_Sup	7.91	2.26	4.8	0.18	.024	.428
4	R. Frontal_Sup	0.00	1.13	-3.81	-0.24	.033	.476
38	R. Hippocampus	1.69	6.78	3.95	0.16	.037	.476
13	L. Frontal_Inf_Tri	2.82	4.52	3.53	0.16	.046	.513
Nodal Efficiency—Biotype 3							
12	R. Frontal_Inf_Oper	0.00	0.00	-6.07	-0.50	.003	.288
61	L. Parietal_Inf	0.79	0.00	4.72	-0.30	.025	.924
7	L. Frontal_Mid	1.57	0.00	-4.22	-0.23	.046	.924
Participation Coefficient—Biotype 1							
9	L. Frontal_Mid_Orb	10.56	2.11	9.57	0.37	<.001	<.001
13	L. Frontal_Inf_Tri	14.79	0.70	12.24	0.45	<.001	<.001
75	**L. Pallidum**	13.38	0.00	10.41	0.40	<.001	<.001
90	R. Temporal_Inf	11.27	0.00	8.45	0.35	<.001	<.001
50	R. Occipital_Sup	7.75	0.70	6.47	0.31	.001	.010
32	R. Cingulum_Ant	6.34	0.00	5.07	0.28	.001	.010
76	R. Pallidum	9.15	0.00	6.33	0.28	.001	.010
4	R. Frontal_Sup	8.45	0.70	6.33	0.28	.001	.014
1	L. Precentral	7.75	0.00	5.49	0.26	.002	.022
17	L. Rolandic_Oper	8.45	0.00	5.63	0.25	.002	.022
65	L. Angular	7.75	0.00	5.21	0.24	.003	.023
64	R. SupraMarginal	8.45	0.00	5.77	0.26	.003	.024
21	L. Olfactory	7.75	0.00	5.35	0.25	.003	.024
45	L. Cuneus	7.04	0.00	4.5	0.22	.010	.063
51	L. Occipital_Mid	7.04	0.70	4.64	0.21	.015	.089
12	R. Frontal_Inf_Oper	7.04	0.00	4.07	0.19	.026	.146
42	R. Amygdala	7.04	0.00	3.93	0.18	.031	.152
68	R. Precuneus	7.75	2.11	4.92	0.19	.031	.152
89	L. Temporal_Inf	8.45	0.00	4.35	0.18	.033	.152
41	L. Amygdala	4.23	2.11	3.66	0.18	.034	.152
24	R. Frontal_Sup_Medial	7.04	0.00	3.93	0.18	.035	.152
35	L. Cingulum_Post	7.75	0.00	4.08	0.18	.046	.189
38	R. Hippocampus	9.86	0.00	4.63	0.18	.049	.189
Participation Coefficient—Biotype 2							
80	R. Heschl	6.78	0.00	4.94	0.25	<.001	.036
19	L. Supp_Motor_Area	7.34	0.00	4.8	0.23	.004	.180
29	L. Insula	2.82	0.00	-5.66	-0.25	.013	.278
72	**R. Caudate**	6.78	0.00	4.1	0.20	.014	.278
8	R. Frontal_Mid	6.78	0.56	4.23	0.19	.017	.278
20	R. Supp_Motor_Area	7.34	0.00	4.09	0.19	.019	.278
69	L. Paracentral_Lobule	7.34	0.00	3.95	0.18	.022	.278
87	L. Temporal_Pole_Mid	2.82	0.00	1.97	0.15	.046	.472
13	L. Frontal_Inf_Tri	5.08	1.69	3.53	0.16	.048	.472
Participation Coefficient—Biotype 3							
40	R. ParaHippocampal	3.15	0.00	7.57	0.29	.002	.162
29	L. Insula	11.81	0.00	-5.32	-0.23	.041	.999

Note: Brain regions with significant case-control difference were shown (group-based permutation testing *p*-value<.05). Biotype 1, 2, and 3 consisted of 142, 177, and 127 ADHD children, respectively.

© 2026 Pan N et al. *JAMA Psychiatry.*

eTable 12. Linear Mixed-effect Models for Longitudinal Changes.

Fixed Effect	Coefficient	Z-value	p-value
Externalizing Problems			
Time in Biotype 1	-2.12	-5.41	<.001
Time in Biotype 2	-3.00	-14.95	<.001
Time in Biotype 3	-2.87	-10.13	<.001
Biotype 1 vs. 2	4.17	1.69	.091
Biotype 1 vs. 3	3.09	1.13	.260
Biotype 2 vs. 3	-1.09	-0.54	.589
Biotype 1 vs. 2 × Time	-0.88	-2.00	.054
Biotype 1 vs. 3 × Time	-0.75	-1.55	.122
Biotype 2 vs. 3 × Time	0.13	0.39	.700
Attention Problems			
Time in Biotype 1	-1.40	-6.45	<.001
Time in Biotype 2	-1.34	-12.05	<.001
Time in Biotype 3	-1.59	-10.12	<.001
Biotype 1 vs. 2	-1.60	-1.57	.116
Biotype 1 vs. 3	-0.51	-0.45	.652
Biotype 2 vs. 3	1.09	1.32	.188
Biotype 1 vs. 2 × Time	0.06	0.24	.812
Biotype 1 vs. 3 × Time	-0.19	-0.70	.481
Biotype 2 vs. 3 × Time	-0.25	-1.28	.200
Deficient Emotional Self-regulation			
Time in Biotype 1	-2.64	-4.76	<.001
Time in Biotype 2	-3.97	-13.23	<.001
Time in Biotype 3	-4.51	-10.49	<.001
Biotype 1 vs. 2	1.64	0.43	.670
Biotype 1 vs. 3	2.42	0.57	.571
Biotype 2 vs. 3	0.78	0.25	.803
Biotype 1 vs. 2 × Time	-1.33	-2.11	**.035**
Biotype 1 vs. 3 × Time	-1.87	-2.66	**.008**
Biotype 2 vs. 3 × Time	-0.54	-1.03	.304

Note: The follow-up data were collected annually over four years at WCH site (exclusively beginning with medication-naïve participants) using the Child Behavior Checklist. The models incorporated time, biotype, and their interaction as fixed effects, with age and sex as covariates. To account for within-subject correlations in repeated measurements, we specified random intercepts for each participant. We fitted the model using the Newton-Raphson method (maximum iterations: 1,000; convergence tolerance: 1×10^{-8}).

© 2026 Pan N et al. *JAMA Psychiatry.*

eTable 13. Medication Usage across Biotypes in Follow-up Samples.

	Daily Dose (mg)	Duration (months)	Cumulative Durg Exposure (mg×months)
Biotype 1	18.75±7.72	9.25±2.60	175.00±87.85
Biotype 2	22.56±10.38	10.24±3.53	251.10±172.22
Biotype 3	20.00±11.92	8.95±3.14	197.25±169.79
H-value	1.78	1.85	3.02
p-value	.411	.398	.221

Note: Data are presented as mean ± standard deviation for follow-up samples (73 subjects) from the WCH site only. These participants were medication-naïve at baseline and subsequently initiated atomoxetine treatment during follow-up. Daily dose is expressed in milligrams per day, duration in months, and cumulative drug exposure as the product of daily dose and treatment duration (mg × months). Kruskal-Wallis analysis was used to examine their differences.

© 2026 Pan N et al. *JAMA Psychiatry.*

eTable 14. Contribution of Cognitive Terms to PLS1 Components.

Term	*Z*-Score	*p* (uncorrected)	*p* (FDR-corrected)
Biotype 1 - PLS1			
Association	6.10	<.001	<.001
Context	5.25	<.001	<.001
Focus	4.93	<.001	<.001
Insight	4.90	<.001	<.001
Utility	4.84	<.001	<.001
Meaning	4.73	<.001	<.001
Learning	4.60	<.001	<.001
Thought	4.58	<.001	<.001
Strategy	4.31	<.001	<.001
Cognitive Control	4.23	<.001	<.001
Detection	3.74	<.001	.002
Strength	3.72	<.001	.002
Adaptation	3.68	<.001	.002
Inhibition	3.58	<.001	.003
Expectancy	3.54	<.001	.003
Sleep	3.37	.001	.006
Induction	3.36	.001	.006
Hyperactivity	3.34	.001	.006
Effort	3.34	.001	.005
Eating	3.32	.001	.006
Response Selection	3.16	.002	.009
Integration	3.09	.002	.011
Risk	3.07	.002	.012
Response Inhibition	3.05	.002	.012
Consciousness	2.95	.003	.015
Loss	2.93	.003	.016
Impulsivity	2.89	.004	.018
Decision	2.83	.005	.020
Arousal	2.82	.005	.021
Valence	2.80	.005	.021
Competition	2.74	.006	.024
Maintenance	2.73	.006	.025
Uncertainty	2.71	.007	.025
Empathy	2.71	.007	.024
Addiction	2.68	.007	.026
Stress	2.67	.008	.026
Encoding	2.63	.009	.028
Anticipation	2.63	.009	.028
Mood	2.62	.009	.027
Monitoring	2.62	.009	.027
Consolidation	2.62	.009	.027
Goal	2.60	.009	.027
Salience	2.57	.010	.029
Memory	2.57	.010	.029
Knowledge	2.55	.011	.029
Intelligence	2.42	.015	.041
Psychosis	2.41	.016	.041
Emotion	2.38	.017	.044
Reinforcement Learning	2.32	.020	.051
Decision Making	2.29	.022	.053
Reward Anticipation	2.14	.033	.078
Emotion Regulation	2.13	.033	.079

© 2026 Pan N et al. *JAMA Psychiatry.*

Anxiety	2.13	.034	.078
Concept	2.12	.034	.077
Search	2.11	.035	.079
Discrimination	2.10	.036	.078
Coordination	2.10	.036	.077
Retention	2.07	.038	.081
Reasoning	2.00	.045	.094
Interference	1.99	.046	.095
Biotype 2 - PLS1			
Recall	-3.61	<.001	.037
Concept	-3.35	.001	.051
Visual Fluency	-3.18	.001	.061
Action	-3.17	.002	.047
Arousal	3.11	.002	.046
Interference	2.96	.003	.063
Manipulation	2.95	.003	.055
Distraction	2.94	.003	.050
Association	2.93	.003	.046
Planning	-2.83	.005	.057
Autobiographical Memory	-2.82	.005	.053
Rule	2.77	.006	.057
Salience	-2.76	.006	.054
Judgment	2.75	.006	.053
Stress	-2.73	.006	.052
Extinction	-2.64	.008	.063
Categorization	-2.62	.009	.063
Retrieval	2.58	.010	.068
Memory Retrieval	2.56	.011	.068
Impulsivity	-2.48	.013	.080
Mental Imagery	-2.42	.015	.090
Familiarity	-2.33	.020	.111
Word Recognition	2.33	.020	.106
Response Selection	2.26	.024	.123
Anticipation	2.14	.032	.160
Strategy	2.14	.033	.155
Object Recognition	-2.09	.037	.167
Rehearsal	-2.07	.038	.168
Naming	-2.02	.044	.186
Psychosis	2.00	.045	.185
Biotype 3 - PLS1			
Adaptation	-5.16	<.001	<.001
Manipulation	-4.32	<.001	.001
Attention	-4.09	<.001	.002
Efficiency	-3.98	<.001	.002
Detection	-3.97	<.001	.002
Integration	-3.89	<.001	.002
Response Selection	-3.87	<.001	.002
Maintenance	-3.82	<.001	.002
Discrimination	-3.73	<.001	.003
Interference	-3.66	<.001	.003
Effort	-3.65	<.001	.003
Skill	-3.63	<.001	.003
Task Difficulty	-3.60	<.001	.003
Monitoring	-3.57	<.001	.003
Action	-3.49	<.001	.004

© 2026 Pan N et al. *JAMA Psychiatry.*

Localization	-3.45	.001	.004
Rehearsal	-3.42	.001	.005
Working Memory	-3.38	.001	.005
Coordination	-3.22	.001	.008
Meaning	-3.11	.002	.012
Focus	-3.08	.002	.012
Inhibition	-3.08	.002	.012
Planning	-2.96	.003	.017
Search	-2.93	.003	.017
Naming	-2.89	.004	.019
Selective Attention	-2.88	.004	.019
Categorization	-2.75	.006	.027
Balance	-2.73	.006	.028
Mental Imagery	-2.68	.007	.031
Sustained Attention	-2.68	.007	.030
Response Inhibition	-2.67	.008	.030
Expectancy	-2.63	.008	.032
Consciousness	-2.62	.009	.033
Empathy	-2.60	.009	.034
Perception	-2.55	.011	.038
Association	-2.54	.011	.038
Goal	-2.47	.014	.045
Imagery	-2.44	.015	.047
Context	-2.44	.015	.046
Updating	-2.43	.015	.046
Competition	-2.39	.017	.051
Reading	-2.34	.019	.056
Utility	-2.33	.020	.057
Strategy	-2.33	.020	.056
Language	-2.27	.023	.064
Visual Attention	-2.25	.024	.065
Spatial Attention	-2.24	.025	.066
Distraction	-2.19	.029	.074
Motor Control	-2.19	.029	.072
Expertise	-2.14	.033	.080
Rhythm	-2.07	.038	.092
Cognitive Control	-2.05	.040	.094
Verbal Fluency	-2.04	.041	.095
Visual Fluency	-2.03	.042	.096
Communication	-2.01	.044	.099
Strength	-2.00	.045	.099
Movement	-2.00	.045	.098
Concept	-2.00	.046	.097
Rule	-1.97	.049	.102

Note: Only terms suprathresholding the p<.05 were presented.

© 2026 Pan N et al. *JAMA Psychiatry.*

eTable 15. Between-biotype Differences in Validation Cohort.

No. AAL	Label of Brain Region	Extreme Deviations in Biotype 1 (%)	Extreme Deviations in Biotype 2 (%)	Extreme Deviations in Biotype 3 (%)	χ^2 statistic	*p*-value	*p*-value (FDR)
Degree Centrality							
87	L. Temporal_Pole_Mid	2.31	1.96	7.34	9.04	.011	.713
25	L. Frontal_Med_Orb	9.25	5.39	2.26	8.11	.017	.713
65	L. Angular	8.09	9.8	2.82	7.48	.024	.713
Nodal Efficiency							
25	L. Frontal_Med_Orb	9.83	5.39	1.13	12.92	.002	.141
57	L. Postcentral	10.4	4.9	4.52	6.34	.042	.909
Participation Coefficient							
40	R. ParaHippocampal	7.51	3.92	18.64	24.78	<.001	<.001
75	L. Pallidum	10.4	3.92	1.13	16.55	<.001	.011
82	R. Temporal_Sup	8.67	3.43	1.13	12.64	.002	.048
26	R. Frontal_Med_Orb	9.83	6.86	1.13	12.29	.002	.048
17	L. Rolandic_Oper	6.36	2.45	0.56	10.29	.006	.092
44	R. Calcarine	9.83	3.92	2.82	9.78	.008	.092
80	R. Heschl	2.89	8.33	2.26	9.64	.008	.092
64	R. SupraMarginal	5.2	2.45	0.00	9.62	.008	.092
71	L. Caudate	2.89	6.86	1.13	9.10	.011	.106
72	R. Caudate	2.31	6.86	1.69	8.45	.015	.132
7	L. Frontal_Mid	2.89	6.37	1.13	7.86	.020	.138
48	R. Lingual	6.36	1.96	1.69	7.76	.021	.138
90	R. Temporal_Inf	6.36	1.96	1.69	7.76	.021	.138
56	R. Fusiform	8.09	2.45	3.39	7.68	.022	.138
81	L. Temporal_Sup	5.78	2.45	1.13	6.78	.034	.199
11	L. Frontal_Inf_Oper	2.89	3.92	0.00	6.69	.035	.199

Note: Biotype 1, 2, and 3 consisted of 173, 204, and 177 ADHD children, respectively. Extreme deviations were defined as deviations of topological patterns exceeding the threshold of Z=|2.0|.

© 2026 Pan N et al. *JAMA Psychiatry.*

eTable 16. Biotypes-specific Extreme Deviation Patterns in Validation Sample.

No. AAL	Brain Region	Extreme Positive Deviations (%)	Extreme Negative Deviations (%)	*p*-value	*p*-value (FDR)
Degree Centrality—Biotype 1					
70	R. Paracentral_Lobule	2.31	5.20	.001	.063
84	R. Temporal_Pole_Sup	4.62	3.47	.001	.063
35	L. Cingulum_Post	4.05	4.62	.006	.168
83	L. Temporal_Pole_Sup	6.36	2.89	.014	.221
58	R. Postcentral	5.20	4.05	.015	.221
53	L. Occipital_Inf	4.62	3.47	.017	.221
65	L. Angular	5.20	2.89	.017	.221
25	L. Frontal_Med_Orb	1.73	7.51	.023	.261
21	L. Olfactory	4.05	2.31	.037	.363
38	R. Hippocampus	4.05	4.62	.041	.363
2	R. Precentral	9.83	0.00	.048	.363
57	L. Postcentral	4.62	4.62	.048	.363
Degree Centrality—Biotype 2					
84	R. Temporal_Pole_Sup	0.98	8.82	<.001	<.001
65	L. Angular	4.90	4.90	.001	.045
81	L. Temporal_Sup	6.86	3.92	.002	.054
13	L. Frontal_Inf_Tri	4.90	3.92	.003	.054
83	L. Temporal_Pole_Sup	4.41	5.39	.003	.054
78	R. Thalamus	3.92	5.39	.007	.105
15	L. Frontal_Inf_Orb	1.47	8.33	.008	.108
27	L. Rectus	5.88	2.94	.016	.182
28	R. Rectus	3.92	5.39	.018	.182
33	L. Cingulum_Mid	0.00	8.33	.031	.281
35	L. Cingulum_Post	5.88	0.98	.036	.291
50	R. Occipital_Sup	0.98	0.49	.039	.291
21	L. Olfactory	2.45	3.43	.048	.335
Degree Centrality—Biotype 3					
84	R. Temporal_Pole_Sup	8.47	5.65	<.001	<.001
83	L. Temporal_Pole_Sup	8.47	3.39	.001	.027
15	L. Frontal_Inf_Orb	4.52	6.21	.003	.072
53	L. Occipital_Inf	6.21	2.82	.004	.072
78	R. Thalamus	5.65	4.52	.005	.072
35	L. Cingulum_Post	6.21	2.26	.005	.072
81	L. Temporal_Sup	4.52	5.65	.006	.072
58	R. Postcentral	5.08	4.52	.010	.110
71	L. Caudate	0.56	9.04	.018	.175
28	R. Rectus	4.52	5.08	.019	.175
13	L. Frontal_Inf_Tri	1.69	5.65	.022	.182
72	R. Caudate	0.56	7.34	.047	.356
Nodal Efficiency—Biotype 1					
84	R. Temporal_Pole_Sup	4.62	2.89	.002	.144
70	R. Paracentral_Lobule	4.62	3.47	.004	.171
35	L. Cingulum_Post	5.20	2.31	.006	.174

© 2026 Pan N et al. *JAMA Psychiatry.*

25	L. Frontal_Med_Orb	2.89	6.94	.018	.324
21	L. Olfactory	4.05	4.05	.018	.324
58	R. Postcentral	4.62	3.47	.030	.393
32	R. Cingulum_Ant	6.36	2.31	.031	.393
4	R. Frontal_Sup	5.78	3.47	.044	.442
83	L. Temporal_Pole_Sup	7.51	2.89	.044	.442
Nodal Efficiency—Biotype 2					
84	R. Temporal_Pole_Sup	0.98	7.84	<.001	<.001
21	L. Olfactory	3.92	6.37	<.001	.018
44	R. Calcarine	4.90	4.41	.015	.312
28	R. Rectus	4.41	4.90	.020	.312
35	L. Cingulum_Post	5.39	0.98	.022	.312
29	L. Insula	0.98	0.49	.030	.312
6	R. Frontal_Sup_Orb	3.43	4.90	.034	.312
81	L. Temporal_Sup	4.41	4.41	.039	.312
53	L. Occipital_Inf	5.39	2.94	.039	.312
36	R. Cingulum_Post	0.98	6.37	.040	.312
65	L. Angular	3.92	3.43	.041	.312
13	L. Frontal_Inf_Tri	2.94	4.41	.042	.312
Nodal Efficiency—Biotype 3					
84	R. Temporal_Pole_Sup	7.34	3.95	<.001	<.001
58	R. Postcentral	5.08	5.65	<.001	.018
53	L. Occipital_Inf	7.34	3.39	.001	.030
70	R. Paracentral_Lobule	5.08	3.39	.002	.036
28	R. Rectus	5.08	5.65	.002	.043
35	L. Cingulum_Post	5.08	2.82	.004	.057
83	L. Temporal_Pole_Sup	9.04	2.26	.014	.175
85	L. Temporal_Mid	3.39	5.65	.026	.272
36	R. Cingulum_Post	2.26	5.65	.027	.272
25	L. Frontal_Med_Orb	0.00	1.13	.030	.274
15	L. Frontal_Inf_Orb	3.95	5.08	.047	.383
Participation Coefficient—Biotype 1					
27	L. Rectus	7.51	5.78	<.001	<.001
44	R. Calcarine	9.83	0.00	<.001	<.001
53	L. Occipital_Inf	9.83	0.00	<.001	<.001
57	L. Postcentral	2.89	10.98	<.001	<.001
58	R. Postcentral	7.51	2.31	<.001	<.001
75	L. Pallidum	10.40	0.00	<.001	<.001
78	R. Thalamus	5.78	4.62	<.001	<.001
29	L. Insula	0.00	0.00	<.001	<.001
3	L. Frontal_Sup	4.05	4.05	<.001	.004
4	R. Frontal_Sup	7.51	1.73	<.001	.004
12	R. Frontal_Inf_Oper	7.51	2.31	.001	.005
82	R. Temporal_Sup	8.67	0.00	.001	.009
20	R. Supp_Motor_Area	5.78	2.89	.002	.014
76	R. Pallidum	8.09	0.00	.002	.014
26	R. Frontal_Med_Orb	2.89	6.94	.002	.014
30	R. Insula	0.00	0.00	.003	.016

© 2026 Pan N et al. *JAMA Psychiatry.*

32	R. Cingulum_Ant	3.47	1.73	.003	.018
46	R. Cuneus	5.78	0.00	.006	.031
6	R. Frontal_Sup_Orb	4.62	0.00	.008	.037
79	L. Heschl	5.78	0.00	.008	.037
42	R. Amygdala	5.78	1.73	.009	.039
86	R. Temporal_Mid	8.09	0.58	.010	.040
8	R. Frontal_Mid	5.20	2.31	.011	.045
43	L. Calcarine	8.09	0.58	.013	.047
36	R. Cingulum_Post	0.00	0.00	.013	.047
73	L. Putamen	4.05	2.89	.014	.049
34	R. Cingulum_Mid	2.31	0.00	.016	.055
38	R. Hippocampus	10.40	0.00	.019	.061
84	R. Temporal_Pole_Sup	1.16	0.00	.022	.068
39	L. ParaHippocampal	6.94	0.00	.030	.090
18	R. Rolandic_Oper	6.94	0.00	.031	.090
90	R. Temporal_Inf	6.36	0.00	.035	.098
10	R. Frontal_Mid_Orb	5.78	0.58	.036	.098
17	L. Rolandic_Oper	6.36	0.00	.037	.098
48	R. Lingual	5.78	0.58	.044	.113
Participation Coefficient—Biotype 2					
38	R. Hippocampus	13.24	0.00	<.001	<.001
53	L. Occipital_Inf	10.78	0.00	<.001	<.001
57	L. Postcentral	2.45	9.80	<.001	<.001
78	R. Thalamus	2.94	5.39	<.001	<.001
80	R. Heschl	8.33	0.00	<.001	<.001
29	L. Insula	0.00	0.00	<.001	<.001
58	R. Postcentral	6.37	2.45	<.001	.002
73	L. Putamen	3.43	6.37	<.001	.002
20	R. Supp_Motor_Area	5.88	3.43	.001	.006
30	R. Insula	0.00	0.00	.001	.013
69	L. Paracentral_Lobule	9.31	0.00	.002	.015
79	L. Heschl	5.88	0.00	.004	.033
72	R. Caudate	6.86	0.00	.007	.047
84	R. Temporal_Pole_Sup	0.98	0.00	.008	.050
12	R. Frontal_Inf_Oper	5.88	0.98	.011	.067
3	L. Frontal_Sup	2.45	3.43	.013	.075
36	R. Cingulum_Post	0.49	0.00	.020	.100
25	L. Frontal_Med_Orb	4.90	1.96	.020	.100
46	R. Cuneus	4.41	0.00	.032	.139
5	L. Frontal_Sup_Orb	4.41	0.00	.033	.139
71	L. Caudate	6.86	0.00	.034	.139
21	L. Olfactory	0.00	0.00	.034	.139
41	L. Amygdala	4.41	1.47	.036	.139
68	R. Precuneus	1.47	0.00	.045	.170
Participation Coefficient—Biotype 3					
40	R. ParaHippocampal	18.64	0.00	<.001	<.001
53	L. Occipital_Inf	15.25	0.00	<.001	<.001
57	L. Postcentral	1.13	10.73	<.001	<.001

© 2026 Pan N et al. *JAMA Psychiatry.*

78	R. Thalamus	2.82	9.60	<.001	<.001
74	R. Putamen	0.00	10.73	<.001	.004
58	R. Postcentral	2.26	6.78	<.001	.006
29	L. Insula	0.00	0.00	.001	.010
32	R. Cingulum_Ant	1.13	4.52	.001	.014
30	R. Insula	0.00	0.00	.002	.020
54	R. Occipital_Inf	4.52	0.00	.002	.020
38	R. Hippocampus	11.30	0.00	.003	.023
34	R. Cingulum_Mid	2.82	0.00	.003	.024
36	R. Cingulum_Post	0.00	0.00	.004	.030
68	R. Precuneus	0.00	0.00	.005	.035
6	R. Frontal_Sup_Orb	4.52	0.00	.011	.066
73	L. Putamen	0.00	6.78	.016	.090
37	L. Hippocampus	5.65	0.00	.017	.090
84	R. Temporal_Pole_Sup	1.13	0.00	.020	.102
11	L. Frontal_Inf_Oper	0.00	0.00	.031	.145
8	R. Frontal_Mid	2.82	3.95	.032	.145
66	R. Angular	0.00	0.00	.035	.145
64	R. SupraMarginal	0.00	0.00	.035	.145
28	R. Rectus	0.56	0.00	.041	.158
5	L. Frontal_Sup_Orb	4.52	0.00	.042	.158
14	R. Frontal_Inf_Tri	0.56	0.00	.044	.158
7	L. Frontal_Mid	1.13	0.00	.048	.165

Note: Only brain regions with significant case-control difference were shown (*p*-value<.05, and the *p*-values were computed based on group-based permutation testing). Biotype 1, 2, and 3 consisted of 173, 204, and 177 ADHD children in validation sample, respectively. Extreme deviations were defined as deviations of topological patterns exceeding the threshold of Z=|2.0|.

© 2026 Pan N et al. *JAMA Psychiatry.*
