# Supplementary material for: Mapping ADHD Heterogeneity and Biotypes by Topological Deviations in Morphometric Similarity Networks
Source: JAMA Psychiatry. 2026 Feb 25;83(5):478–90. doi: 10.1001/jamapsychiatry.2026.0001 (PMC12936971; doi:10.1001/jamapsychiatry.2026.0001)
Supplement: Supplement 2. — Data Sharing Statement. [file jamapsychiatry-e260001-s002.pdf]

Data Sharing Statement

Pan. Mapping ADHD Heterogeneity and Biotypes by Topological Deviations in Morphometric Similarity Networks. *JAMA Psychiatry*. Published February 25, 2026.
doi:10.1001/jamapsychiatry.2026.0001

Data
Data available: Yes
Data types: Deidentified participant data
How to access data: UC and WCH datasets are available upon reasonable request with approval from the respective principal investigators [Drs. R.K.M. and M.P.D. for UC, delbelmp@ucmail.uc.edu; Drs. Y. Chen and Q.G. for WCH, chenying85285@163.com] due to data policy and ethical restrictions.
When available: With publication

Supporting Documents
Document types: Statistical/analytic code
How to access documents: All associated code and pre-trained models to reproduce the analysis results of normative models, fusion models, prediction of HYDRA clustering, and neural contextualization will be openly available at https://osf.io/wjgsr/ upon publication of the article.
When available: With publication

Additional Information
Who can access the data: Researchers whose proposed use of the data has been approved.
Types of analyses: UC and WCH datasets are available upon reasonable request with approval from the respective principal investigators.
Mechanisms of data availability: After approval of a proposal.
